# The Fate of Glyphosate and AMPA in a Freshwater Endorheic Basin: An Ecotoxicological Risk Assessment

**DOI:** 10.3390/toxics6010003

**Published:** 2017-12-21

**Authors:** Rocío Inés Bonansea, Iohanna Filippi, Daniel Alberto Wunderlin, Damián José Gabriel Marino, María Valeria Amé

**Affiliations:** 1 Facultad de Ciencias Químicas, Dto. Bioquímica Clínica—CIBICI (Centro de Investigaciones en Bioquímica Clínica e Inmunología), Universidad Nacional de Córdoba—CONICET, Haya de la Torre esq. Medina Allende, 5000-Córdoba, Argentina; rociobonansea@hotmail.com (R.I.B.); chani_fi@hotmail.com (I.F.); 2 Facultad de Ciencias Químicas, Dto. Química Orgánica—ICYTAC (Instituto de Ciencia y Tecnología de Alimentos Córdoba), Universidad Nacional de Córdoba—CONICET, Av. Juan Filloy s/n, Ciudad Universitaria, 5000 Córdoba, Argentina; dwunder@fcq.unc.edu.ar; 3 Facultad de Ciencias Exactas, CIMA (Centro de Investigaciones del Medio Ambiente), Universidad Nacional de La Plata, Calle 115 esq. 47, 1900-La Plata, Argentina; damian.marino@gmail.com

**Keywords:** herbicide, metabolite, environmental fate, hazard quotient, risk assessment

## Abstract

Glyphosate is the most widely used herbicide worldwide. However, there are some uncertain aspects with respect to its environmental fate. To evaluate the existence and distribution of this pesticide and its metabolite, aminomethylphosphonic acid (AMPA), their presence in fresh water, sediment, and suspended particulate matter (SPM) was measured in samples collected in a river running across a large city and through areas with intensive and extensive agriculture. The aquatic risk associated to the occurrence of these compounds was estimated using the hazard quotient (HQ) calculation for water and sediment. From the analyzed samples, overall 35% contained glyphosate, AMPA, or both compounds. Concentrations of the analytes were spread in different percentages depending on the environmental matrices considered, with levels ranging from 12 to 20 times higher for glyphosate and AMPA in sediment and SPM, as compared with the levels found in water. The most polluted area was situated within a green belt zone of the city; while in second place were sites located in areas of extensive agriculture. Aquatic organisms inhabiting areas both inside and outside agricultural areas are threatened by water glyphosate concentrations. Benthic organisms inside the greenbelt zone and inside the lower basin are threatened by the concentrations of glyphosate in sediment. Even when the concentrations measured in water were below the levels of concern for wildlife, results showed the risk of agricultural practices to aquatic biota. An update of the limits established for freshwater biota protection is needed.

## 1. Introduction

In South America, the extensive production of cereals and oilseeds for the international market coexists with intensive horticulture and family farming, involving wide geographical distribution (mainly close to urban centers), and a broad variety of cultivated species. The extensive agricultural model, based on glyphosate-resistant transgenic soybean farming, no-till, and the intensive use of fertilizers and pesticides, is highly dependent on modern technologies [1,2]. In contrast, intensive crops such as fruits and vegetables are characterized by high labor demand per unit of output. Typically, this is a small-scale activity performed by peasant family production units, with all their members participating [3,4]. Usually, in the greenbelts of big cities, the staggered mode in which a diverse range of crops are grown allows farmers to cultivate a large number of crops in small plots, leading to a higher frequency of pesticide application. Therefore, there is a heavy burden of pesticides in two scenarios: in extensive crops, due to the extensive areas sprayed, and in horticultural crops, due to the process of spraying throughout the year. This also implies significant environmental pollution, with approximately 47% of the product deposited in adjacent soils and waters or dispersed in the atmosphere [5].

Glyphosate is a non-selective herbicide and is the main pesticide used in Argentina, where approximately 281 million liters of glyphosate are employed per year [6]. However, there are still some uncertain aspects with respect to the understanding of its environmental fate, since this depends on climatic conditions, geological features, and other factors such as the formula and presentation of the product as well as the application technique. This herbicide is a polar, highly water soluble substance (11.6 g L^−1^, 25 °C), which are features that favor the pollution of the aquatic system. Even though its persistence is relatively short compared to other pesticides (it has a half-life that ranges from 2 to 91 days), when it is combined with soil particles or sediment, glyphosate persists for longer and it may last up to 215 days [7]. The microbial degradation product of glyphosate, aminomethylphosphonic acid (AMPA), is considered the most important metabolite. Aminomethylphosphonic acid is more persistent than glyphosate, with a higher half-life ranging from 76 to 240 days in soil [8]. Due to the current widespread use of glyphosate, there are multiple routes through which pollution of the aquatic environment may occur, mostly through drift during application or surface runoff after application [9]. These phenomena may result in significant quantities of glyphosate and AMPA being found in freshwater resources [10]. This fact has been probed by different studies carried out in several countries [11,12,13,14]. These findings have alerted the community to such a degree that the latest reviews have focused on their effects on human health and aquatic environmental risks. Risk studies taking into account predicted environmental concentrations conducted by Giesy et al. ([15], pp. 35–104), Solomon and Thompson ([10], pp. 289–324), and Williams et al. ([16], pp. 117–165), concluded that the use of glyphosate implies no risk for aquatic biota and humans. Moreover, the United States Environmental Protection Agency [17] classifies glyphosate formulations as low or non-toxic to birds and mammals, and they are designated as being from practically non-toxic to moderately toxic to aquatic invertebrates. In contrast, when the studies were done according to measured environmental concentrations, they showed that there they pose a significant risk to aquatic microorganisms, aquatic invertebrates, and amphibians [8]. Recently, the World Health Organization classified the herbicide glyphosate as probably carcinogenic to humans (group 2A) [18].

In Argentina, concentrations of glyphosate and AMPA have been measured in fresh water from few areas of the country: Buenos Aires, and the Corrientes and Entre Ríos provinces [19,20,21,22]. Furthermore, ecological risk assessments due to the presence of pesticides in aquatic environments are scarce. Peluso et al. ([23], pp. 1177–1199) assessed the environmental risk of organochlorine pesticides following a model known as DelAzulPestRisk, though the presence of glyphosate was not included in calculations. On account of the actual situation of pollution in Argentina, as well as in other South American countries, we considered it relevant to include other areas with intensive and extensive agricultural models where glyphosate use is widespread, and use an approach that evaluates risk assessment by the calculation of a hazard quotient (HQ) taking into account the ratio between the measured environmental concentrations (MECs) and the predicted no-effect concentration (PNEC) to reduce some uncertainties as regards the probability of risk to aquatic biota [24].

Therefore, the first aim of this study was to evaluate the aquatic environmental presence and distribution of glyphosate and AMPA in Suquía River; in order to discuss the environmental fate of glyphosate and AMPA in areas with intensive and extensive agricultural models, their concentrations in different compartments (water, sediments, and suspended particulate matter, SPM) were quantified. The second aim of this study was to estimate the environmental risk associated to the presence of glyphosate and AMPA in this aquatic environment in areas with intensive and extensive agricultural models.

## 2. Materials and Methods

### 2.1. Chemical and Reagents

Glyphosate (99.2%) and AMPA (99%) reference standards, isotope-labelled glyphosate (GLY*, 1,2-^13^C, ^15^N, used as an internal standard (IS)), and derivatizing agent 9-fluorenmethylcholoroformate (FMOC-Cl), were purchased from Sigma-Aldrich (St. Louis, MO, USA). Analytical reagent-grade potassium dihydrogen phosphate, ammonium acetate, and disodium tetraboratedecahydrate were obtained from J.T. Baker (Philipsburg, NJ, USA). Dichloromethane was of pesticide residual grade, and was also purchased from J.T. Baker (Philipsburg, NJ, USA). Methanol and acetonitrile were of HPLC grade and were also obtained from J.T Baker (Philipsburg, NJ, USA). Ultra-pure water was prepared using a Milli-Q water purification system from Millipore (Bedford, MA, USA).

Standard stock solutions of glyphosate and AMPA were prepared by dissolving the exact weight of high-purity substances in borate buffer solution 25 mM (pH = 9).

### 2.2. Apparatus

The HPLC-ESI-MS equipment consisted of a binary-pump Agilent 1100™ system pump (Agilent Technologies Inc., Miami, FL, USA) in tandem with a mass-quadrupole VL mass spectrometer with an electrospray ionization source (ESI Agilent Technologies Inc., Miami, FL, USA). A Rheodyne 7725i injector with a 20-µL loop was used. The liquid chromatography separation was performed on a C-18 X-Select™ column (75 mm × 4.6 mm and 3 µm pore size, from Waters Corp., Milford, MA, USA) kept at 25 ± 1 °C, at a flow rate of 0.4 mL min^−1^ with a gradient of water (A) and methanol (B) with 5 mM ammonium acetate as mobile phase. The initial conditions were 20% B for 0.5 min, and then the gradient was increased to 80% B in 2.5 min and kept isocratic for 4 min. Subsequently, the gradient was increased again to 95% B in 1 min and kept isocratic for 1 min. Finally, the gradient was returned to initial conditions. Selected ion monitoring (SIM) mode was used to obtain the maximum sensitivity for quantitative analysis. Two significant ions from each analyte were chosen for quantification: Glyphosate-FMOC 390 and 168 m/z, AMPA-FMOC 332 and 110, GLY*-FMOC 392 and 170 [25].

### 2.3. Study Area and Sampling

**Figure 1:**
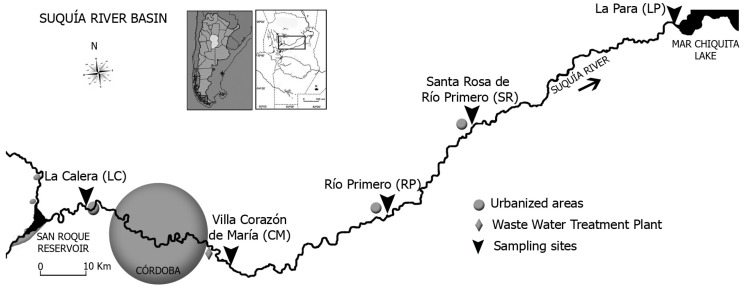
Study sites in the Suquía River basin (Córdoba–Argentina).

The Suquía River basin is located in a semi-arid region of the province of Córdoba (Argentina; Figure 1). The river drainage area covers approximately 7700 km^2^, and the basin represents the main drinking water source for Cordoba city. It also serves for recreation and some sport fishing. Suquía River begins at the San Roque dam; 35 km downstream it flows for about 40 km across Córdoba city (1.3 million inhabitants). Near the eastern edge of the city, the river receives the city sewage discharge and then continues across an agricultural production area up to Mar Chiquita Lake (150 km downstream). Five sampling sites along this river were selected to understand the spatial distribution of pesticides concentration in the river system and compare areas with different land use including intensive and extensive crops (Figure 1, Table 1). The first site was located upstream of Córdoba city, in a mountainous area without agricultural activities (La Calera, LC). Then, downstream of Córdoba city, the second site was placed in Villa Corazón de María (CM), nearly 13 km after the wastewater treatment plant (WWTP) of Córdoba city. The CM site is located in an area where vegetables are grown to supply the city, using an intensive agricultural model. Then, in the lower basin and along an extensive crops cultivated area, three sites were selected: Río Primero (RP), Santa Rosa de Río Primero (SR), and La Para (LP; Table 1).

Study sites were sampled twice during low and high application periods of pesticides, during the years 2010–2011 on four sampling dates: July 2010 (low), November 2010 (high), April 2011 (low), and June 2011 (high).

Two water samples were collected in 15 mL polypropylene tubes at 20–30 cm below the river surface; they were filtered immediately after sampling through 0.45-µm nylon membrane (Millipore, MA, USA) to separate the water from the SPM. Duplicated sediment samples were collected in 50 mL polypropylene tubes at the same sites of the water samples. All the samples were stored in polypropylene tubes at −20 °C until analysis.

### 2.4. Physical and Chemical Parameters

Water temperature, dissolved oxygen, pH, and conductivity were measured in the field using a multiparametric equipment, previously calibrated in the laboratory (Multiline F/Set 3, WTW, Weilheim, Bavaria, Germany; [26]). Texture, pH, and conductivity were determined in sediment samples, according to the Soil Science Society of America methodology [27] while organic matter (OM) was measured by wet combustion [28].

### 2.5. Glyphosate and AMPA Analyses

#### 2.5.1. Water Samples

A representative sub-sample of filtered water (1 mL) was derivatized by adding 50 µL of 400 mM borate buffer adjusted to pH = 9 followed by 1 mL of FMOC–Cl solution (1 mg mL^−1^), and was left overnight in darkness at room temperature [29]. After that, samples were filtered through a 0.22 µm nylon membrane and were injected into the high-performance liquid chromatography coupled to mass spectrometry system (HPLC-ESI-MS, Agilent Technologies Inc., Miami, FL, USA). Prior to derivatization, GLY*, at a final concentration of 100 µg L^−1^, was added to the water samples.

#### 2.5.2. Sediments and Suspended Particulate Matter Samples

A representative sub-sample of sediments (8 g) and suspended particulate matter (contained in 1 L river water), were overloaded with 1:5 of GLY* solution and left for 30 min in order to stabilize the system. After that, samples were extracted with 25 mL of potassium dihydrogen phosphate solution by shaking and sonication [22]. Then, these reaction tubes were centrifuged, and 1 mL of resulting supernatants was derivatizated as in water and injected into the HPLC-ESI-MS equipment.

#### 2.5.3. Analytical Method

The sensitivity and linearity of the HPLC-ESI-MS method was validated preparing standards of glyphosate and AMPA in concentrations ranging from 1 to 1000 µg L^−1^ containing 100 µg L^−1^ of GLY*.

Fortified samples of water, sediments, and SPM with glyphosate, AMPA, and GLY* at 100 µg L^−1^, 500 µg kg^−1^, and 0.5 µg L^−1^, respectively, were also analyzed for recovery experiments. Results obtained from five replicates were satisfactory (between 70% and 106%). The limits of detection (LODs) and quantification (LOQs) of the method were experimentally estimated as the concentration of analyte giving a signal-to-noise ratio of 3 and 10, respectively [30]. The limits obtained in water samples were LOD = 0.5 µg L^−1^ and LOQ = 1 µg L^−1^, while in sediments and SPM the values were LOD = 3 µg kg^−1^ and LOQ = 10 µg kg^−1^.

By the performed extraction, a satisfying solution was found for the high organic matter content in the sediment, reducing the matrix effect to 10% for glyphosate and 25% for AMPA, facilitating the quantification in lower levels.

### 2.6. Statistics

Environmental data were tested for normality and homogeneity of variance. Since the pesticide contents did not meet these assumptions, the Kruskal–Wallis test [31] was performed followed using Dunn’s multiple comparison test. Differences were considered significant at *p* < 0.05. Cluster analysis was performed using the average linkage method with Euclidean distance. Statistical analyses were performed using the statistical package, STATISTICA 8 from StatSoft Inc (Palo Alto, CA, USA).

### 2.7. Ecotoxicological Risk Assessment

To estimate the potential risk to non-target aquatic species in the present study, the HQ of glyphosate and AMPA were calculated as the ratio between the MEC and PNEC.

The highest concentration of glyphosate and AMPA measured in the water and sediment samples of each site in Suquía River basin were considered as the MEC. The PNEC values were calculated according to the European Technical Guidance Document on Risk Assessment (ETGDRA) [32]. Therefore, to calculate PNEC for glyphosate and AMPA in water, we identified the lowest no observed effects concentration (NOEC) and applied an assessment factor of 10. According to ETGDRA [30] the use of this factor is appropriate when long-term toxicity NOECs are available from at least three species across three trophic levels (e.g., fish, *Daphnia*, and algae). To calculate PNECs for glyphosate in sediments, NOEC values for benthic organisms were considered and an assessment factor of 100 was applied. An assessment factor of 100 applies on the lowest NOEC derived in long-term tests with a relevant test organism. The AMPA PNEC in sediments could not be evaluated because data for benthic organisms were unavailable.

To derive PNEC values, the no observed effects concentration (NOEC), median effective concentration (EC50), median inhibitory concentration (IC50), or median lethal concentration (LC50) obtained from toxicity tests reported by Giesy et al. ([15], pp. 35–104) and updated by Annett et al. ([8], pp. 458–479) were taken into account. The considered toxicity data of glyphosate and AMPA for freshwater aquatic microorganisms, invertebrates, fish, amphibians, and macrophytes are listed in Supplementary Material (Tables S1–S6). This information was obtained from exposure studies with glyphosate of technical grade and different formulations of glyphosate-based herbicides. Thus, conversions to acid equivalents (µg a.e.L^−1^) have been made to simplify direct comparison of exposure or fate data [15].

## 3. Results and Discussion

### 3.1. Water and Sediments Physical and Chemical Characteristics

Results obtained by means of water quality parameters and sediments chemical characteristics at all sampling sites are shown in Table 2. The analyzed parameters varied significantly from site to site. Conductivity, dissolved oxygen, and pH measured in water samples show that Córdoba city impacts negatively on water quality. Other studies performed in the Suquía River basin proved that the WWTP of Córdoba city, as well as the runoff from the city and the cultivated fields located near the river are significant in terms of pollutant input [33,34,35,36]. According to texture analysis, sediments were classified in line with the United State Department of Agricultural system. As such, CM and SR were categorized as sandy, LC and RP as sandy-silty, and LP as silty. The OM % was significantly higher in LC because of the contribution from the river bank vegetation. The sediment pH ranged between 6.6 and 7.5 and the conductivity between 578.5 and 922.5; both measurements are similar to those reported by Merlo et al. ([37], pp. 5034–5045).

### 3.2. Occurrence of Glyphosate and AMPA

Levels of the herbicide glyphosate and its metabolite AMPA were measured in fresh water, sediment, and SPM samples from the Suquía River basin (Córdoba, Argentina). This monitoring program was carried out over 1 year (between 2010 and 2011), with sampling in five sites located along the river (Figure 1), making a total of 60 samples. From all the analyzed samples, 35% contained glyphosate, AMPA, or both compounds (Table 3). Glyphosate was detected at least once in samples collected in all the studied sites, whereas AMPA was detected at least once in four sites. In the LC site, the AMPA concentration was always below the LOD. The presence of the herbicide and/or its metabolite in all five sites is proof of its use all along the basin. This detection frequency is similar to that of the rural and urban freshwater surface in Ontario [38]. With regard to the concentration of glyphosate and AMPA spread in the different environmental matrices studied, 61% was found in sediment, with glyphosate levels ranging from <LOD to 1882.3 µg kg^−1^ and AMPA levels ranging from <LOD to 266.1 µg kg^−1^. In total, 36% of glyphosate and AMPA was measured in SPM, with the range for glyphosate being between <LOD and 1570.7 µg kg^−1^ and for AMPA from <LOD to 684.9 µg kg^−1^. Only 3% of the total measured glyphosate and AMPA was in the water compartment, with levels ranging from <LOD to 125.0 µg L^−1^ for glyphosate and from <LOD to 4.8 µg L^−1^ for AMPA. Despite the high solubility of glyphosate and AMPA in water, our results show a high affinity of these compounds for SPM and sediment in contrast to water (12 and 20 times higher, respectively) [39]. In order to explain the partition of pesticide between both matrices, a pesticide sediment-runoff partition coefficient (Kp) expressed as L kg^−1^ was calculated. In the case of CM and RP, the Kp was 287 and 24 L kg^−1^, respectively, denoting a high adsorption on sediments. Lupi et al. ([40], pp. 687–694) reported similar results for glyphosate in Quequén Grande river (26 L kg^−1^). The higher concentrations of glyphosate and AMPA found in sediment when compared to water could be also explained by the fact that these pollutants have lower possibilities for microbial decomposition when they are adsorbed to particulate matter [41].

Glyphosate was detected in water samples at a maximum of 125.0 µg L^−1^, which is equivalent to 166.7 µg L^−1^ glyphosate isopropylamine salt. Thus measured levels did not exceed the limits established for freshwater aquatic protection (240 µg L^−1^) according to the Argentinean Environmental Water Quality Guidelines [42].

**Figure 2:**
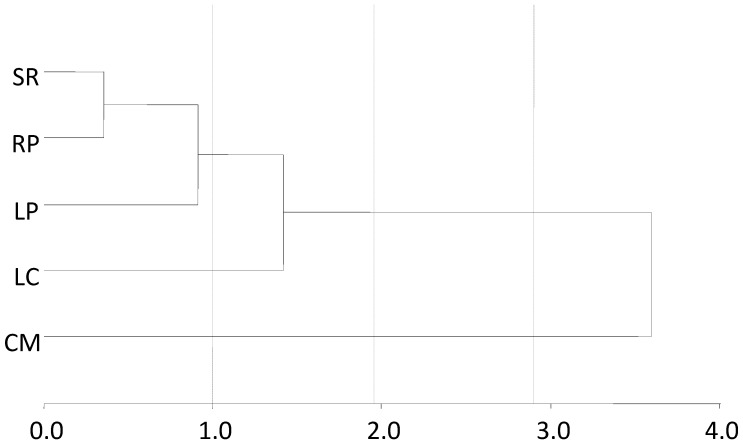
Tree diagram obtained by cluster analysis of glyphosate and aminomethylphosphonic acid (AMPA) quantified in water, sediment and suspended particulate matter samples from the Suquía River basin, using the average linkage method with Euclidean distance. LC: La Calera, CM: Villa Corazón de María, RP: Río Primero, SR: Santa Rosa de Río Primero, LP: La Para.

The tree diagram obtained by cluster analysis of glyphosate and AMPA concentrations in fresh water, sediment, and SPM, identified differences between the sampling sites (Figure 2). It shows two clusters at 50% of the total distance, wherein the lower cluster represents only the CM site. At 25% of the total distance, three clusters are observed. The top clusters represent SR, RP, and LP, while LC follows, and lastly, there is CM. Thus, a high similarity among the three sites with extensive agricultural models can be observed, with less similarity to LC, the site without agricultural activities, and a clear difference to CM, the location with intensive crops.

**Figure 3:**
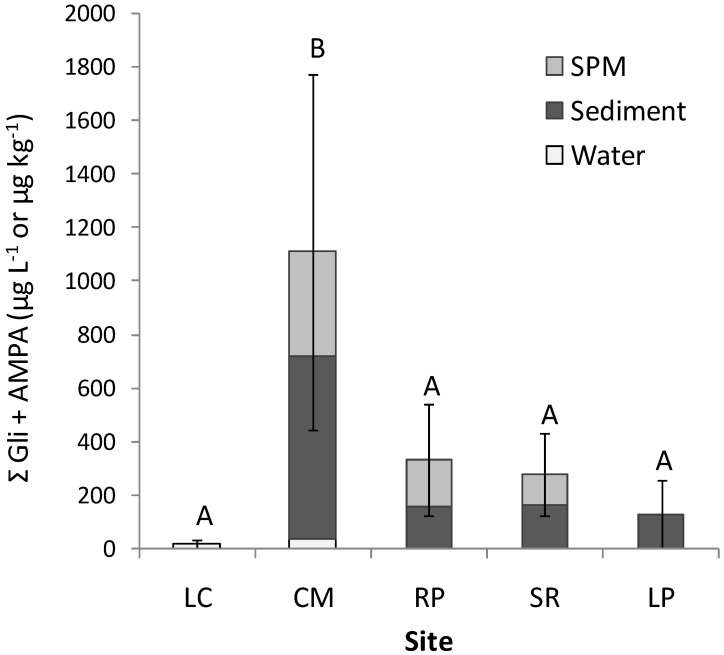
Addition of glyphosate and aminomethylphosphonic acid (AMPA) quantified in water (µg L^−1^), sediment (µg kg^−1^) and suspended particulate matter (SPM µg kg^−1^) samples from the Suquía River basin. Different letters indicate significant differences between monitoring sites (*p* < 0.05). LC: La Calera, CM: Villa Corazón de María, RP: Río Primero, SR: Santa Rosa de Río Primero, LP: La Para.

The concentrations found at each site reflect that the most polluted sample location is CM (Figure 3; Kruskall–Wallis *p* < 0.05), with a maximum level of glyphosate plus AMPA of 127.2 µg L^−1^ in water, 2148.4 µg kg^−1^ in sediment, and 1570.7 µg kg^−1^ in SPM, displaying a 50% detection frequency. The measured concentrations could be the result of the intensive use of this pesticide on the neighboring vegetable gardens. Even without significant differences, the sites that showed an intermediate contamination rate were RP, SR, and LP. The former had a maximum level of glyphosate plus AMPA of 4.8 µg L^−1^ in water, 390.9 µg kg^−1^ in sediment, and 684.9 µg kg^−1^ in SPM, with a highest detection frequency result of 58%. In SR there was an absence of glyphosate and AMPA in water, with a maximum value of glyphosate plus AMPA of 335.4 µg kg^−1^ in sediment and 473.5 µg kg^−1^ in SPM, with a detection frequency of 42%. In LP neither glyphosate nor AMPA was found in water and SPM, but there was a maximum value of glyphosate plus AMPA of 472.1 µg kg^−1^ in sediment, with a detection frequency of 17%. These three sites are situated in the main agriculture area of Córdoba state, where the cultivated sectors reach the riverbank.

The least polluted area was LC. In this site a maximum value of 70.0 µg L^−1^ of glyphosate was measured in water, but the herbicide and its metabolite were absent in both SPM and sediments, with detection frequency of 8%, the lowest percentage in the Suquía River basin. However, the presence of glyphosate in the water of LC could be attributed to point sources, such as the cleaning of machinery on the riverbank, discarding of recipients with herbicide leftovers, or sporadic domestic usage, since no agricultural activities can be registered in the area (Table 1).

Similar concentrations of glyphosate and AMPA were found in other agricultural areas of Argentina, such as in the northwest of the state of Buenos Aires [20]. However, the concentrations here reported were higher than those measured in the southeast of the same province [19]. Our results showed also higher concentrations of glyphosate and AMPA in water and SPM than those found in studies carried out in Paraná river and Mesopotamic Pampas agroecosystem, as reported by Ronco et al. ([22], pp. 771–779) and Primost ([21], pp. 771–779), respectively.

The concentration of glyphosate in relation to its metabolite AMPA indicates that time had passed from the moment the herbicide was employed [43]. According to the results found in the Suquía River basin, in the LC site glyphosate was detected only in water and it can be assumed the contamination was recent due to the fact its metabolite was not found. In CM, the pollution was mainly produced by glyphosate and, in addition, it was spread in all the analyzed matrices. This fact would indicate a constant glyphosate runoff into the ecosystem.

In RP and SR, the highest concentration was that of the metabolite AMPA, with a greater percentage found in SPM and sediments. This situation could indicate that certain time had passed since the application of the chemical on those sites. In LP, it was observed that the highest concentration was present in the form of glyphosate, which indicates recent contamination, and besides, every positive measure of both the herbicide and its metabolite was found in the sediment. This outcome can be understood if the texture results of the sediment are taken into account. Of all the studied sites, the higher percentage of clay was found in LP, a component known to have a high affinity with the herbicide [44]. With regards to the temporal distribution, during the present study no significant differences were found between low and high application periods (data not shown).

The horticultural productivity in the subtropical regions of the world is severely limited by pests and diseases affecting crops. As a result, the losses in the field and the reduction of the commercial values of the products make the horticultural business less profitable than expected. The fact that the quality of the products has become a priority worldwide has led to the generation of a group of quality standards in response to the demands of the consuming market. The main criterion used regarding this issue is related to the visual aspect related to shape, color, and the absence of damage [45]. The use of agrochemicals is the most common method used for the control of pests, diseases, and the improvement in the quality of the products. Based on our results, the use of glyphosate in intensive crops provokes more pollution to the watercourse than the extensive crops.

### 3.3. Ecological Risk Assessment

In order to estimate the environmental risk associated to the presence of glyphosate and AMPA in areas with intensive and extensive crops, the MECs at five sampling points located along Suquía River and the PNECs obtained from the most sensitive species approach were used for the calculation of HQ (Table 4). The HQ values in LC and CM indicate that there is significant risk to aquatic organisms associated with the presence of glyphosate in water. In a similar manner, the HQs obtained considering the herbicide content in sediments and the effects over benthic organisms were of 6.7 and 1.4 in CM and LP respectively, suggesting risk in sediment dwellers. HQs over 1 were calculated by Annett et al. ([8], pp. 458–479) for fish species, using an MEC in a surface stream of Argentina [20], and for aquatic microorganisms, invertebrates, and amphibians considering realistic environmental exposure concentrations in forest wetland measured by Thompson et al. ([46], pp. 843–849). Benthic organisms in RP and SR would not be at risk of toxic effects due to the presence of glyphosate in sediments, since none of the calculated HQs reached the risk significance level of 1.

The HQs obtained for AMPA derived from available toxicity data in aquatic organisms and AMPA levels in fresh water suggest it is not a dangerous metabolite. However, there few toxicity studies of AMPA in aquatic organisms on which to base this assessment. Furthermore, it was not possible to calculate HQs in sediment since no toxicity data was available for this metabolite in benthic organisms.

The results of hazard assessment for glyphosate show that both aquatic and benthic organisms of the Suquía River basin are at risk in areas where intensive agricultural models are applied. Extensive crops seem to be dangerous only to benthic species. The presence of glyphosate in unexpected areas due to point sources of pollution also endangers aquatic species.

## 4. Conclusions

Our study showed a high occurrence of glyphosate in water, sediment, and SPM all along the Suquía River basin, indicating the wide use of this herbicide in the area close to the watercourse. The higher concentrations were associated to an intensive agricultural model applied in the greenbelt zone of Córdoba city. In this area both aquatic and benthic organisms may be at risk. Those sampling sites located in the low basin of the river, where extensive crops are grown, were not only polluted with glyphosate but also with its metabolite AMPA, revealing a continuous application of the herbicide for extended periods of time. However, only the concentration of glyphosate in sediment of LP points out a threat to benthic organisms. The samples taken in LC, a town on the hillside hence without agricultural activity, also presented high concentrations of glyphosate in water in an isolated event. This episode may be because of the lack of awareness with regards to chemical disposal and the cleaning of application equipment, tanks, and containers. The hazard assessment for glyphosate in water at this site implies that effects in aquatic organisms may occur. With respect to AMPA, there is no risk associated to its presence in the Suquía River basin. However, the lack of toxicity data on benthic species prevents us from calculating the risk of measured concentrations in sediments.

The presence of glyphosate and its metabolite in aquatic environments requires stricter control, and further studies on the potential toxic effects of glyphosate and AMPA on the non-target native aquatic organisms are necessary.

## Figures and Tables

**Table 1:** Land uses (percentage) in a 5-km radius of sampling sites in the Suquía River basin, 2010.

Site	% of Urban Construction	% of Industries	% of Agriculture	% of Native Vegetation	% of River	Agricultural Model
**LC**	42.2	6.7	0.0	46.0	4.8	None
**CM**	0.1	0.2	55.0	33.0	11.6	Intensive
**RP**	12.3	4.0	66.0	3.4	14.2	Extensive
**SR**	30.4	1.0	50.5	7.4	10.8	Extensive
**LP**	0.0	0.0	73.8	21.6	4.6	Extensive

**Table 2:** Water quality parameters measured in situ and sediment chemical characteristics at each sampling site on the Suquía River basin (mean ± standard deviation). Different letters, when indicated, represent significant differences between monitoring stations (*p* < 0.05).

Site	Water				Sediments					
	DO (mg L^−1^)	CE (µS cm^−1^)	pH	Temp. (°C)	OM %	CE (µS cm^−1^)	pH	Texture		
								Sand %	Silt %	Clay %
**LC**	^C^ 10.1 ± 2.5	^A^ 268 ± 28	^B^ 8.1 ± 0.2	17.2 ± 7.0	^B^ 8.4 ± 4.7	988.0 ± 754.0	^A^ 6.6 ± 0.2	41.3 ± 23.3	53.3 ± 23.0	5.5 ± 3.4
**CM**	^A^ 3.2 ± 1.7	^C^ 1388 ± 156	^A^ 7.5 ± 0.2	20.7 ± 8.3	^AB^ 5.0 ± 4.0	578.5 ± 226.9	^AB^ 6.9 ± 0.3	54.2 ± 22.4	40.0 ± 25.2	5.3 ± 4.2
**RP**	^B^ 6.9 ± 1.3	^B^ 1106 ± 248	^AB^ 7.7 ± 0.2	20.5 ± 9.2	^AB^ 5.6 ± 2.1	740.3 ± 225.5	^B^ 6.9 ± 0.3	36.0 ± 21.5	63.0 ± 21.8	2.5 ± 0.5
**SR**	^BC^ 8.8 ± 2.0	^B^ 992 ± 302	^AB^ 8.0 ± 0.4	20.4 ± 7.7	^A^ 2.4 ± 2.0	922.5 ± 657.4	^BC^ 7.0 ± 0.5	56.5 ± 6.9	39.0 ± 4.6	4.5 ± 5.7
**LP**	^BC^ 8.7 ± 1.6	^B^ 1253 ± 69	^C^ 8.5 ± 0.2	20.1 ± 6.9	^A^ 3.5 ± 2.7	825.5 ± 535.9	^C^ 7.5 ± 0.5	17.0 ± 7.1	74.8 ± 5.2	8.3 ± 5.3

DO: dissolved oxygen, CE: Conductivity, Temp: temperature, OM: organic matter, LC: La Calera, CM: Villa Corazón de María, RP: Río Primero, SR: Santa Rosa de Río Primero, LP: La Para.

**Table 3:** Concentration of glyphosate and aminomethylphosphonic acid (AMPA) in water, sediment and suspended particulate matter (SPM) samples collected in Suquía River basin. Different letters, when indicated, represent significant differences between monitoring stations (*p* < 0.05).

Site		Glyphosate			AMPA		
		Water	Sediments	SPM	Water	Sediments	SPM
		(µg L^−1^)	(µg kg^−1^)	(µg kg^−1^)	(µg L^−1^)	(µg kg^−1^)	(µg kg^−1^)
**LC**	Mean	17.5	^A^ <LOD	<LOD	<LOD	^A^ <LOD	<LOD
	Min	<LOD	<LOD	<LOD	<LOD	<LOD	<LOD
	Max	70.0	<LOD	<LOD	<LOD	<LOD	<LOD
**CM**	Mean	35.2	^B^ 615.4	392.7	0.6	^AB^ 66.5	<LOD
	Min	<LOD	<LOD	<LOD	<LOD	<LOD	<LOD
	Max	125.0	1882.3	1570.7	2.2	266.1	<LOD
**RP**	Mean	<LOD	^AB^ 61.9	<LOD	2.1	^C^ 97.0	171.2
	Min	<LOD	<LOD	<LOD	<LOD	38.5	<LOD
	Max	<LOD	168.7	<LOD	4.8	222.2	684.9
**SR**	Mean	<LOD	^B^ 89.5	<LOD	<LOD	^BC^ 73.0	118.4
	Min	<LOD	23.1	<LOD	<LOD	23.9	<LOD
	Max	<LOD	139.0	<LOD	<LOD	196.4	473.5
**LP**	Mean	<LOD	^AB^ 105.1	<LOD	<LOD	^AB^ 22.6	<LOD
	Min	<LOD	<LOD	<LOD	<LOD	<LOD	<LOD
	Max	<LOD	381.9	<LOD	<LOD	90.2	<LOD

Min: minimum, Max: maximum, LOD: limit of detection, SPM: suspended particulate matter, LC: La Calera, CM: Villa Corazón de María, RP: Río Primero, SR: Santa Rosa de Río Primero, LP: La Para.

**Table 4:** Hazard quotient (HQ) of glyphosate and aminomethylphosphonic acid (AMPA) in water and sediments of the Suquía River basin.

Site		Glyphosate		AMPA
		Aquatic Organisms	Benthic Organisms	Aquatic Organisms
		PNEC = 23 µg a.e. L^−1^	PNEC = 280 µg a.e. kg^−1^	PNEC = 790 µg a.e. L^−1^
**LC**	MEC	70 µg a.e. L^−1^	<LOD	<LOD
	HQ	3.0	N/A	N/A
**CM**	MEC	125 µg a.e. L^−1^	1882.3 µg kg^−1^	2.2 µg a.e. L^−1^
	HQ	5.4	6.7	3.0E-03
**RP**	MEC	<LOD	168.7 µg kg^−1^	222.2 µg a.e. L^−1^
	HQ	N/A	0.6	0.3
**SR**	MEC	<LOD	139.0 µg kg^−1^	<LOD
	HQ	N/A	0.5	N/A
**LP**	MEC	<LOD	381.9 µg kg^−1^	<LOD
	HQ	N/A	1.4	N/A

N/A: not applicable, PNEC: predicted no effect concentration, HQ: hazard quotient, MECs: measured environmental concentrations, LC: La Calera, CM: Villa Corazón de María, RP: Río Primero, SR: Santa Rosa de Río Primero, LP: La Para.

## Supplementary Materials

The following are available online at www.mdpi.com/2305-6304/6/1/3/s1. Table S1: Toxicity of glyphosate and AMPA to aquatic microorganisms; Table S2: Toxicity of glyphosate and AMPA to aquatic invertebrates; Table S3: Toxicity of glyphosate and AMPA to fish; Table S4: Toxicity of glyphosate and AMPA to amphibians; Table S5: Toxicity of glyphosate and AMPA to aquatic macrophytes; Table S6: Toxicity of glyphosate to benthic organisms.

Supplementary File 1
