# Supplementary material for: The Fate of Glyphosate and AMPA in a Freshwater Endorheic Basin: An Ecotoxicological Risk Assessment
Source: Toxics. 2017 Dec 21;6(1):3. doi: 10.3390/toxics6010003 (PMC5874776; doi:10.3390/toxics6010003)
Supplement: Supplementary File 1 [file toxics-06-00003-s001.pdf]

<u>**Supplementary material**</u>

This supplement contains:

- **Table S1**: Toxicity of Glyphosate and AMPA to aquatic microorganisms.
- **Table S2**: Toxicity of Glyphosate and AMPA to aquatic invertebrates.
- **Table S3**: Toxicity of Glyphosate and AMPA to fish.
- **Table S4**: Toxicity of Glyphosate and AMPA to amphibians.
- **Table S5**: Toxicity of Glyphosate and AMPA to aquatic macrophytes.
- **Table S6**: Toxicity of Glyphosate to benthic organisms.
- **References of supplementary material.**

Table S1: Toxicity of Glyphosate and AMPA to aquatic microorganisms.

AQUATIC MICROORGANISMS									
**Glyphosate**									
		**IC50 / EC50**			**NOEC**				
**Species**	**Test duration**	**(mg a.i L^{-1})**	**(mg RU L^{-1})**	**(mg a.e L^{-1})**	**(mg a.i L^{-1})**	**(mg RU L^{-1})**	**(mg a.e L^{-1})**	**Formulation**	**Reference**
Anabaena flos-aquae	7 d			15			9.7	Glyphosate	Malcolm Pirnie, 1987a
Ankistrodesmus sp.	4 d	412		309	20		15.0	Glyphosate (tested as IPA salt)	Gardner et al., 1997
Chlamydomonas eugametos	4 d			> 169			16.9	Glyphosate	Hess, 1980
Chlorella pyrenoidosa	4 d			3.53				Glyphosate (TC: technical product)	Ma, 2002
Chlorella pyrenoidosa	4 d			590				Glyphosate	Maule and Wright, 1984
Chlorella pyrenoidosa	7 d		189	58.6				Roundup®	Hernando et al., 1989
Chlorella saccharophila	3 d			26				Glyphosate (Analytical Standard)	Vendrell et al., 2009
Chlorella sorokiniana	4 d		3	0.9		2	0.6	Roundup®	Christy et al., 1981
Chlorella vulgaris	3 d			24.5				Glyphosate (Analytical Standard)	Vendrell et al., 2009
Chlorococcum hypnosporum	4 d			68				Glyphosate	Maule and Wright, 1984
Navicula pelliculosa	7 d			42			33.6	Glyphosate	Malcolm Pirnie, 1987c
Scenedesmus acutus	4 d			10.2			2	Glyphosate	Saenz et al., 1997
Scenedesmus acutus	3 d			40.6				Glyphosate (Analytical Standard)	Vendrell et al., 2009
Scenedesmus oblicuos	4 d			55.85				Glyphosate (TC: technical product)	Ma, 2002
Scenedesmus quadricauda	4 d			7.2			0.77	Glyphosate	Saenz et al., 1997
Scenedesmus subspicatus	3 d			41.7				Glyphosate (Analytical Standard)	Vendrell et al., 2009
Scenedesmus subspicatus	3 d	72.9		54.7	10.5		7.88	Glyphosate (tested as IPA salt)	Dengler and Mende, 1994b
Scenedesmus subspicatus	3 d	166		125	26.4		19.80	Glyphosate (tested as IPA salt)	Dengler and Mende, 1994b
Selenastrum capricomutum	4 d		2.6	0.8				Roundup®	Thomas et al., 1990
Selenastrum capricomutum	3 d		2.1	0.7		0.73	**0.23**	Roundup®	LISEC, 1989
Selenastrum capricomutum	3 d		8	2.5				Roundup®	LISEC, 1989
Selenastrum capricornutum	4 d			5.81				Roundup®	Tsui and Chu, 2003
Selenastrum capricornutum	4 d			24.7				Glyphosate Technical grade	Tsui and Chu, 2003
Selenastrum capricornutum	4 d			41				Glyphosate (tested as IPA salt)	Tsui and Chu, 2003
Selenastrum capricornutum	4 d			21.8 or 26.3				Glyphosate	Bozeman et al., 1989
Selenastrum capricornutum	3 d			485			45	Glyphosate	NATEC, 1990

AQUATIC MICROORGANISMS									
Glyphosate									
Species	Test duration	IC50 / EC50 (mg a.i L^{-1})	IC50 / EC50 (mg RU L^{-1})	IC50 / EC50 (mg a.e L^{-1})	NOEC (mg a.i L^{-1})	NOEC (mg RU L^{-1})	NOEC (mg a.e L^{-1})	Formulation	Reference
Selenastrum capricornutum	7 d			13.8			10.6	Glyphosate	Malcolm Pimie, 1987b
Tetrahymena pyriformis	40 h			29.5				Roundup®	Tsui and Chu, 2003
Tetrahymena pyriformis	40 h			648				Glyphosate Technical grade	Tsui and Chu, 2003
Tetrahymena pyriformis	40 h			386				Glyphosate (tested as IPA salt)	Tsui and Chu, 2003
Tetrahymena pyriformis	9 h			>500				Glyphosate (Fluka)	Bonnet et al., 2007

AMPA					
Species	Test Duration	IC50 / EC50 (mg AMPA L^{-1})	NOEC (mg AMPA L^{-1})	Formulation	Reference
Scenedesmus subspicatus	3 d	90	7,9	AMPA (Analytical Standard)	Dengler and Mende, 1994a
Tetrahymena pyriformis	9 h	>5000		AMPA (Analytical Standard, Fluka)	Bonnet, 2007

IC50: median inhibitory concentration, EC50: median effective concentration, NOEC: no observed effects concentration, RU: Roundup. Bold value indicates lower NOEC for aquatic organisms.

Table S2: Toxicity of Glyphosate and AMPA to aquatic microorganisms.

**AQUATIC INVERTEBRATES**										
**Glyphosate**										
			**LC50 / EC50**			**NOEC**				
**Species**	**Life stage**	**Test duration**	**(mg a.i L^{-1})**	**(mg RU L^{-1})**	**(mg a.e L^{-1})**	**(mg a.i L^{-1})**	**(mg RU L^{-1})**	**(mg a.e L^{-1})**	**Formulation**	**Reference**
Anopheles quadrimaculatus	Larvae	1 d		673.4	208.8				Roundup®	Holck and Meek, 1987
Ceriodaphnia dubia		2 d			5.39				Roundup®	Tsui and Chu 2003
Ceriodaphnia dubia		2 d			147				Glyphosate Technical grade	Tsui and Chu 2003
Ceriodaphnia dubia		2 d			415				Glyphosate (tested as IPA salt)	Tsui and Chu, 2003
Daphnia magna		2 d		24	7		7.8	2.4	Roundup®	EG & G Bionomics, 1980f
Daphnia magna		2 d		12.9	4.0		4.6	1.4	Roundup®	EG & G Bionomics, 1980e
Daphnia magna		2 d			780			560	Glyphosate (tested as acid)	ABC Inc., 1978a
Daphnia magna		2 d	930		698	320		240	Glyphosate (tested as IPA salt)	ABC Inc., 1981a
Daphnia magna		21 d					3.2	1.0	Roundup®	ABC Inc., 1989b
Daphnia magna		21 d						100	Glyphosate	ABC Inc., 1989c
Daphnia magna		21 d						50	Glyphosate	ABC Inc., 1982d
Daphnia magna		2 d		9.7	3.0				Roundup®	Folmar et al., 1979
Daphnia pulex		2 d		19	6				Roundup®	Hartman and Martin, 1984
Daphnia pulex		4 d		25.5	7.9				Roundup®	Servizi et al., 1987
Gammarus pseudolimnaeus		2 d		42	13		4.4	**1.4**	Roundup®	ABC Inc., 1982b
Gammarus pseudolimnaeus		2 d		200	62				Roundup®	Folmar et al., 1979
Gammarus pseudolimnaeus		4 d		138.7	43.0				Roundup®	Folmar et al., 1979
Hyalella azteca		10 d	>530		397.5	265		199	Glyphosate (tested as IPA salt)	Beyers, 1993
Lampsilis siliquiodea	Glochidia	24 h			3				Roundup®	Bringolf et al., 2007
Lampsilis siliquiodea	Glochidia	48 h			2.9				Roundup®	Bringolf et al., 2007
Lampsilis siliquiodea	Juvenile	48 h			5.9				Roundup®	Bringolf et al., 2007
Lampsilis siliquiodea	Juvenile	96 h			5.9				Roundup®	Bringolf et al., 2007
Lampsilis siliquiodea	Juvenile	7 d			>7.4				Roundup®	Bringolf et al., 2007
Lampsilis siliquiodea	Juvenile	14 d			>7.4				Roundup®	Bringolf et al., 2007
Lampsilis siliquiodea	Juvenile	21 d			6				Roundup®	Bringolf et al., 2007

AQUATIC INVERTEBRATES

Glyphosate

Species	Life stage	Test duration	LC50 / EC50			NOEC			Formulation	Reference
			(mg a.i L^{-1})	(mg RU L^{-1})	(mg a.e L^{-1})	(mg a.i L^{-1})	(mg RU L^{-1})	(mg a.e L^{-1})		
Lampsilis siliquiodea	Juvenile	28 d			3.7				Roundup®	Bringolf et al., 2007
Lampsilis siliquiodea	Glochidia	24 h			>148				Aqua Star (active ingredient: 53.8% glyphosate IPA salt)	Bringolf et al., 2007
Lampsilis siliquiodea	Glochidia	48 h			>148				Aqua Star (active ingredient: 53.8% glyphosate IPA salt)	Bringolf et al., 2007
Lampsilis siliquiodea	Juvenile	48 h			>148				Aqua Star (active ingredient: 53.8% glyphosate IPA salt)	Bringolf et al., 2007
Lampsilis siliquiodea	Juvenile	96 h			>148				Aqua Star (active ingredient: 53.8% glyphosate IPA salt)	Bringolf et al., 2007
Lampsilis siliquiodea	Juvenile	7 d			>148				Aqua Star (active ingredient: 53.8% glyphosate IPA salt)	Bringolf et al., 2007
Lampsilis siliquiodea	Juvenile	14 d			>148				Aqua Star (active ingredient: 53.8% glyphosate IPA salt)	Bringolf et al., 2007
Lampsilis siliquiodea	Juvenile	21 d			957.3				Aqua Star (active ingredient: 53.8% glyphosate IPA salt)	Bringolf et al., 2007
Lampsilis siliquiodea	Juvenile	28 d			43.8				Aqua Star (active ingredient: 53.8% glyphosate IPA salt)	Bringolf et al., 2007
Lampsilis siliquiodea	Glochidia	24 h			>200				Glyphosate (technical grade)	Bringolf et al., 2007
Lampsilis siliquiodea	Glochidia	48 h			>200				Glyphosate (technical grade)	Bringolf et al., 2007
Lampsilis siliquiodea	Juvenile	48 h			>200				Glyphosate (technical grade)	Bringolf et al., 2007
Lampsilis siliquiodea	Juvenile	96 h			>200				Glyphosate (technical grade)	Bringolf et al., 2007
Lampsilis siliquiodea	Juvenile	7 d			>200				Glyphosate (technical grade)	Bringolf et al., 2007
Lampsilis siliquiodea	Juvenile	14 d			>200				Glyphosate (technical grade)	Bringolf et al., 2007
Lampsilis siliquiodea	Juvenile	21 d			>200				Glyphosate (technical grade)	Bringolf et al., 2007
Lampsilis siliquiodea	Glochidia	24 h			5.9				Glyphosate IPA (technical grade)	Bringolf et al., 2007
Lampsilis siliquiodea	Glochidia	48 h			5				Glyphosate IPA (technical grade)	Bringolf et al., 2007
Lampsilis siliquiodea	Juvenile	48 h			8.3				Glyphosate IPA (technical grade)	Bringolf et al., 2007
Lampsilis siliquiodea	Juvenile	96 h			7.2				Glyphosate IPA (technical grade)	Bringolf et al., 2007
Lampsilis siliquiodea	Juvenile	7 d			7.6				Glyphosate IPA (technical grade)	Bringolf et al., 2007
Lampsilis siliquiodea	Juvenile	14 d			6.9				Glyphosate IPA (technical grade)	Bringolf et al., 2007
Lampsilis siliquiodea	Juvenile	21 d			5.4				Glyphosate IPA (technical grade)	Bringolf et al., 2007
Lampsilis siliquiodea	Juvenile	28 d			4.8				Glyphosate IPA (technical grade)	Bringolf et al., 2007
Lampsilis siliquiodea	Glochidia	24 h			6				Glyphosate (tested as IPA salt)	Bringolf et al., 2007
Lampsilis siliquiodea	Glochidia	48 h			4.6				Glyphosate (tested as IPA salt)	Bringolf et al., 2007

AQUATIC INVERTEBRATES

Glyphosate

Species	Life stage	Test duration	LC50 / EC50			NOEC			Formulation	Reference
			(mg a.i L^{-1})	(mg RU L^{-1})	(mg a.e L^{-1})	(mg a.i L^{-1})	(mg RU L^{-1})	(mg a.e L^{-1})		
Lampsilis siliquiodea	Juvenile	48 h			9.9				Glyphosate (tested as IPA salt)	Bringolf et al., 2007
Lampsilis siliquiodea	Juvenile	96 h			6.3				Glyphosate (tested as IPA salt)	Bringolf et al., 2007
Orconectes nais		4 d		7	2				Roundup®	Mayer and Ellersieck, 1986
Procambarus clarkii		4 d		47.3	14.7				Roundup®	Holck and Meek, 1987
Pseudosuccinea columella		n.r			98.9				Glyphosate (tested as acid)	Thompson, 1989
Scapholeberis kingi		0,125 d		61	19				Roundup®	Sun, 1987

AMPA

Species	Life stage	Test Duration	LC50 / EC50 (mg AMPA L^{-1})	NOEC (mg AMPA L^{-1})	Formulation	Reference
Daphnia magna		2 dias	690	320	AMPA (Analytical Standard)	ABC Inc., 1991a

LC50: median lethal concentration, EC50: median effective concentration, NOEC: no observed effects concentration, RU: Roundup. Bold value indicates lower NOEC for aquatic invertebrates.

Table S3: Toxicity of Glyphosate and AMPA to fish.

FISH										
**Glyphosate**										
**Species**	**Life stage**	**Test duration**	**LC50**			**NOEC**			**Formulation**	**Reference**
			**(mg a.i L^{-1})**	**(mg RU L^{-1})**	**(mg a.e L^{-1})**	**(mg a.i L^{-1})**	**(mg RU L^{-1})**	**(mg a.e L^{-1})**		
Cyprinodon variegatus		4 d			>1000			1000	Glyphosate (tested as acid)	EG & G Bionomics, 1978
Cyprinus carpio		4 d		10	3		5.6	1.7	Roundup®	Liong et al., 1988
Cyprinus carpio		4 d		15	5				Roundup®	Tooby et al., 1980
Cyprinus carpio		4 d		26	8				Roundup®	Sun, 1987
Gambusia affinis		2 d		15	5				Roundup®	Sun, 1987
Hybognathus placitus		4 d	>648		>486	648		486	Glyphosate (tested as IPA salt)	Beyers, 1995
Ictalurus punctatus	Adult	4 d		42	13				Roundup®	Folmar et al., 1979
Ictalurus punctatus	Fry	4 d		10.6	3.3				Roundup®	Folmar et al., 1979
Ictalurus punctatus		4 d		39	12		23	7	Roundup®	EG & G Bionomics, 1980a
Jenynsia multidentata		96h		19.02	5.8962				Roundup®	Hued et al., 2012
**Lepomis macrochirus**		4 d		16.1	5.0				Roundup®	Folmar et al., 1979
Lepomis macrochirus		2 d			>24			24	Glyphosate (tested as acid)	Bionomics, 1973
Lepomis macrochirus		4 d			120			100	Glyphosate (tested as acid)	ABC Inc., 1978c
Lepomis macrochirus		4 d	140-220		105-165				Glyphosate (tested as IPA salt)	Folmar et al., 1979
Lepomis macrochirus		4 d	>1000		>750	560		420	Glyphosate (tested as IPA salt)	ABC Inc., 1981b
Lepomis macrochirus		4 d		34	11		21	7	Roundup®	EG & G Bionomics, 1980b
**Lepomis macrochirus**		4 d		5.8	1.8		2.2	0.7	Roundup®	ABC Inc., 1982a
letalurus punctatus		4 d	130		97.5				Glyphosate (tested as IPA salt)	Folmar et al., 1979
lordanella jloridae	Juvenile	4 d			>30				Glyphosate (tested as acid)	Holdway and Dixon, 1988
Oncorhynchus gorbuscha (hard water)		4 d		14	4				Roundup®	Wan et al., 1989
Oncorhynchus gorbuscha (soft water)		4 d		31	10				Roundup®	Wan et al., 1989
Oncorhynchus gorbuscha(hard water)		4 d			190				Glyphosate (tested as acid)	Wan et al., 1989
Oncorhynchus gorbuscha (soft water)		4 d			23				Glyphosate (tested as acid)	Wan et al., 1989
Oncorhynchus keta (hard water)		4 d		11	3.4				Roundup®	Wan et al., 1989
Oncorhynchus keta (soft water)		4d		19	6				Roundup®	Wan et al., 1989
Oncorhynchus keta (hard water)		4 d			148				Glyphosate (tested as acid)	Wan et al., 1989

FISH										
**Glyphosate**										
**Species**	**Life stage**	**Test duration**	**LC50**			**NOEC**			**Formulation**	**Reference**
			**(mg a.i L^{-1})**	**(mg RU L^{-1})**	**(mg a.e L^{-1})**	**(mg a.i L^{-1})**	**(mg RU L^{-1})**	**(mg a.e L^{-1})**		
Oncorhynchus keta (soft water)		4 d			22				Glyphosate (tested as acid)	Wan et al., 1989
Oncorhynchus kisutch	Fry	4 d		42	13				Roundup®	Servizi et al., 1987
Oncorhynchus kisutch		4 d		22	7				Roundup®	Mitchell et al., 1987
Oncorhynchus kisutch (hard water)		4 d			174				Glyphosate (tested as acid)	Wan et al., 1989
Oncorhynchus kisutch (soft water)		4 d			36				Glyphosate (tested as acid)	Wan et al., 1989
Oncorhynchus kisutch (hard water)		4 d		13	4.0				Roundup®	Wan et al., 1989
Oncorhynchus kisutch (soft water)		4 d		27	8.4				Roundup®	Wan et al., 1989
Oncorhynchus mykiss		4 d		8.2	2.5		6.4	2.0	Roundup®	ABC Inc., 1982c
Oncorhynchus mykiss		4 d		22	7		8	2.5	Roundup®	EG & G Bionomics 1980g
Oncorhynchus mykiss		4 d		27	8		6.75	2.1	Roundup®	Morgan et al., 1991
Oncorhynchus mykiss		4 d		27	8		21.4	6.6	Roundup®	EG & G Bionomics, 1980c
Oncorhynchus mykiss	Fingerling	4 d		4.2-27	1.3-8.4				Roundup®	Folmar et al., 1979
Oncorhynchus mykiss		4 d		52	16				Roundup®	Hildebrand et al., 1982
Oncorhynchus mykiss		4 d		15	5				Roundup®	Mitchell et al., 1987
Oncorhynchus mykiss		4 d		33.6	10.4				Roundup®	Morgan and Kiceniuk, 1992
Oncorhynchus mykiss		4 d			86			42	Glyphosate (tested as acid)	ABC Inc., 1978b
Oncorhynchus mykiss		4 d	>1000		>750	1000		750	Glyphosate (tested as IPA salt)	ABC Inc., 1981c
Oncorhynchus mykiss		4 d	140-240		105-180				Glyphosate (tested as IPA salt)	Folmar et al., 1979
Oncorhynchus mykiss		21 d					2.4	0.744	Roundup®	ABC Inc., 1989d
Oncorhynchus mykiss		21 d						52	Glyphosate	ABC Inc. 1989a
Oncorhynchus mykiss (hard water)		4 d			197				Glyphosate (tested as acid)	Wan et al., 1989
Oncorhynchus mykiss (soft water)		4 d			22				Glyphosate (tested as acid)	Wan et al., 1989
Oncorhynchus mykiss (hard water)		4 d		14	4				Roundup®	Wan et al., 1989
Oncorhynchus mykiss (soft water)		4 d		15	5				Roundup®	Wan et al., 1989
Oncorhynchus nerka		4 d		26.7	8.3				Roundup®	Servizi et al., 1987

FISH										
**Glyphosate**										
**Species**	**Life stage**	**Test duration**	**LC50**			**NOEC**			**Formulation**	**Reference**
			**(mg a.i L^{-1})**	**(mg RU L^{-1})**	**(mg a.e L^{-1})**	**(mg a.i L^{-1})**	**(mg RU L^{-1})**	**(mg a.e L^{-1})**		
Oncorhynchus tshawytsha		4 d		20	6				Roundup®	Mitchell et al., 1987
Oncorhynchus tshawytsha (soft water)		4d		27	8				Roundup®	Wan et al., 1989
Oncorhynchus tshawytsha (hard water)		4 d		17	5				Roundup®	Wan et al., 1989
Oncorhynchus tshawytsha (hard water)		4 d			211				Glyphosate (tested as acid)	Wan et al., 1989
Oncorhynchus tshawytsha (soft water)		4 d			30				Glyphosate (tested as acid)	Wan et al., 1989
Pimephales promelas		4 d		23	7		13.6	4.2	Roundup®	EG & G Bionomics, 198Od
Pimephales promelas		4 d	97		72.75				Glyphosate (tested as IPA salt)	Folmar et al., 1979
Pimephales promelas		255 d						26	Glyphosate	EG & G Bionomics, 1975
Pimephales promelas		4 d		7.4	2.3				Roundup®	Folmar et al., 1979
Pimephales promelas		4 d	>648		>486	648		486	Glyphosate (tested as IPA salt)	Beyers, 1995
Prochilodus lineatus	Juvenile	6 h		20.8	6.5				Roundup®	Langiano and Martinez, 2008
Prochilodus lineatus	Juvenile	24 h		17.3	5.4				Roundup®	Langiano and Martinez, 2008
Prochilodus lineatus	Juvenile	96 h		13.7	4.2				Roundup®	Langiano and Martinez, 2008
Rasbora heteromorpha		4 d			168			<100	Glyphosate (tested as acid)	HRC, 1977
Rhamdia quelen	Fingerlings	96h			7.3				Roundup®	Kreutz et al., 2008
Tilapia sp.		4 d		7.4	2.3		1	**0.31**	Roundup®	Liong et al., 1988

AMPA						
**Species**	**Life stage**	**Test Duration**	**LC50**	**NOEC**	**Formulation**	**Reference**
			**(mg AMPA L^{-1})**	**(mg AMPA L^{-1})**		
Oncorhynchus mykiss		4 d	520	33	AMPA	ABC Inc., 1991b

LC50: median lethal concentration, NOEC: no observed effects concentration, RU: Roundup. Bold value indicates lower NOEC for fish.

Table S4: Toxicity of Glyphosate and AMPA to amphibians.

Species	Life stage	Test duration	LC50 (mg a.i L^{-1})	LC50 (mg RU L^{-1})	LC50 (mg a.e L^{-1})	NOEC (mg a.i L^{-1})	NOEC (mg RU L^{-1})	NOEC (mg a.e L^{-1})	LOEC (mg a.e L^{-1})	Formulation	Reference
**AMPHIBIANS**											
**Glyphosate**											
Ambystoma gracile		96 h			2.8					Roundup Original Max	Relyea and Jones, 2009
Ambystoma laterale		96 h			3.2					Roundup Original Max	Relyea and Jones, 2009
Ambystoma maculatum		96 h			2.8					Roundup Original Max	Relyea and Jones, 2009
**American toads**	Tadpole	16 d	2.52		1.89					Roundup®	Relyea, 2005
Bufo americanus	Larvae	24 h			4.2					Roundup Original	Howe et al., 2004
Bufo americanus	Larvae	96 h			<4					Roundup Original	Howe et al., 2004
Bufo americanus	Embryo	24 h			>8					Roundup Original	Howe et al., 2004
Bufo americanus	Embryo	96 h			8					Roundup Original	Howe et al., 2004
Bufo americanus	Embryo	96 h (pH=6)			4.8					Vision (glyphosate-based herbicide containing a 15% (weight:weight) polyethoxylated tallow amine surfactant blend)	Edginton et al., 2004
Bufo americanus	Embryo	96 h (pH=7.5)			6.4					Vision (glyphosate-based herbicide containing a 15% (weight:weight) polyethoxylated tallow amine surfactant blend)	Edginton et al., 2004
Bufo americanus	Larvae	96 h (pH=6)			2.9					Vision (glyphosate-based herbicide containing a 15% (weight:weight) polyethoxylated tallow amine surfactant blend)	Edginton et al., 2004
Bufo americanus	Larvae	96 h (pH=7.5)			1.7					Vision (glyphosate-based herbicide containing a 15% (weight:weight) polyethoxylated tallow amine surfactant blend)	Edginton et al., 2004
Bufo americanus	Larvae	96 h			1.6					Roundup Original Max	Relyea and Jones, 2009
Bufo boreas	Larvae	96 h			2					Roundup Original Max	Relyea and Jones, 2009
Bufo fowleri	Larvae	96 h			4.21			3.4		Roundup®	Moore et al., 2012
Bufo fowleri	Larvae	96 h			4.21			3.4	3.95	Roundup Original	Fuentes et al., 2011
Bufo fowleri	Larvae	96 h			1.96			1.54	1.56	Roundup WeatherMAX	Fuentes et al., 2011
Crinia insignifera	Tadpole	2 d		10	3					Roundup®	Mann and Bidwell, 1999
Crinia insignifera	Newly emerged	2 d		144	45					Roundup®	Mann and Bidwell, 1999
Crinia insignifera	Adult	2 d		137	42					Roundup®	Mann and Bidwell, 1999
Crinia insignifera	Tadpole	2 d		<54.9	<17					Roundup®	Bidwell and Gorrie, 1995
Crinia insignifera	Adult	4 d		96.8	30.0	54		16.74		Roundup®	Bidwell and Gorrie, 1995
Crinia insignifera	Newly emerged	2 d			83.6					Glyphosate (tested as acid)	Mann and Bidwell, 1999
Crinia insignifera	Adult	4 d			78			45		Glyphosate (tested as acid)	Bidwell and Gorrie, 1995

AMPHIBIANS											
**Glyphosate**											
**Species**	**Life stage**	**Test duration**	**LC50**			**NOEC**			**LOEC**	**Formulation**	**Reference**
			**(mg a.i L^{-1})**	**(mg RU L^{-1})**	**(mg a.e L^{-1})**	**(mg a.i L^{-1})**	**(mg RU L^{-1})**	**(mg a.e L^{-1})**	**(mg a.e L^{-1})**		
Crinia insignifera	Tadpole	2 d	>466		>350					Glyphosate (tested as IPA salt)	Mann and Bidwell, 1999
Heleioporus eyrei	Tadpole	2 d		17.5	5.4					Roundup®	Mann and Bidwell, 1999
Heleioporus eyrei	tadpole	2 d	>373		>279					Glyphosate (tested as IPA salt)	Mann and Bidwell, 1999
Hyla chrysoscelis	Larvae	96 h			2.5			1.74		Roundup®	Moore et al., 2012
Hyla chrysoscelis	Larvae	96h			3.26			2.48	2.87	Roundup WeatherMAX	Fuentes et al., 2011
Hyla chrysoscelis	Larvae	96h			2.5			1.74	2.1	Roundup Original	Fuentes et al., 2011
Hyla versicolor	Larvae	96 h			1.7					Roundup Original Max	Relyea and Jones, 2009
Hyla versicolor	Tadpole	16 d	1.35		1.01					Roundup®	Relyea, 2005
Leopard frogs	Tadpole	16 d	2.46		1.85					Roundup®	Relyea, 2005
Limnodynastes dorsalis	Tadpole	2 d		8.3	2.60					Roundup®	Mann and Bidwell, 1999
Limnodynastes dorsalis	Tadpole	2 d	>400		>300					Glyphosate (tested as IPA salt)	Mann and Bidwell, 1999
Litoria moorei	Tadpole	2 d		8.1	2.5					Roundup®	Mann and Bidwell, 1999
Litoria moorei	Tadpole	2 d		32.2	10.0					Roundup®	Mann and Bidwell, 1999
Litoria moorei	Tadpole	4 d		18.7	5.8	5.5		1.705		Roundup®	Bidwell and Gorrie, 1995
Litoria moorei	Adult	4 d		>165	>51	165		51		Roundup®	Bidwell and Gorrie, 1995
Litoria moorei	Tadpole	2 d			121					Glyphosate (tested as acid)	Mann and Bidwell, 1999
Litoria moorei	Tadpole	2 d			81.2					Glyphosate (tested as acid)	Mann and Bidwell, 1999
Litoria moorei	Tadpole	4 d			111					Glyphosate (tested as acid)	Bidwell and Gorrie, 1995
Litoria moorei	Adult	4 d			>180			180		Glyphosate (tested as acid)	Bidwell and Gorrie, 1995
Litoria moorei	Tadpole	2 d	>343		>257					Glyphosate (tested as IPA salt)	Mann and Bidwell, 1999
Notophthalmus viridescens		96 h			2.7					Roundup Original Max	Relyea and Jones, 2009
Pseudacris crucifer	Larvae	96 h			0.8					Roundup Original Max	Relyea and Jones, 2009
Rana cascadae	Larvae	96 h			1.7					Roundup Original Max	Relyea and Jones, 2009
Rana catesbeiana	Larvae	96 h			2.77			2.02		Roundup®	Moore et al., 2012
Rana catesbeiana	Larvae	96 h			0.8					Roundup Original Max	Relyea and Jones, 2009
Rana catesbeiana	Larvae	96 h			2.77			2.02	2.52	Roundup Original	Fuentes et al., 2011
Rana catesbeiana	Larvae	96h			1.97			1.33	1.37	Roundup WeatherMAX	Fuentes et al., 2011
Rana clamitans	Larvae	24 h			2					Roundup Original	Howe et al., 2004
Rana clamitans	Larvae	96 h			2					Roundup Original	Howe et al., 2004
Rana clamitans	Embryo	24 h			>8					Roundup Original	Howe et al., 2004
Rana clamitans	Embryo	96 h			7.1					Roundup Original	Howe et al., 2004
Rana clamitans	Larvae	24 h			>17.9					Roundup Biactive	Howe et al., 2004
Rana clamitans	Larvae	96 h			>17.9					Roundup Biactive	Howe et al., 2004
Rana clamitans	Larvae	24 h			>17.9					Touchdown	Howe et al., 2004
Rana clamitans	Larvae	96 h			>17.9					Touchdown	Howe et al., 2004
Rana clamitans	Larvae	24 h			>17.9					Glyfos BIO	Howe et al., 2004
Rana clamitans	Larvae	96 h			>17.9					Glyfos BIO	Howe et al., 2004
Rana clamitans	Larvae	24 h			9					Glyfos AU	Howe et al., 2004
Rana clamitans	Larvae	96 h			8.9					Glyfos AU	Howe et al., 2004

AMPHIBIANS											
**Glyphosate**											
**Species**	**Life stage**	**Test duration**	**LC50**			**NOEC**			**LOEC**	**Formulation**	**Reference**
			**(mg a.i L^{-1})**	**(mg RU L^{-1})**	**(mg a.e L^{-1})**	**(mg a.i L^{-1})**	**(mg RU L^{-1})**	**(mg a.e L^{-1})**	**(mg a.e L^{-1})**		
Rana clamitans	Larvae	24 h			2.3					Roundup Transorb	Howe et al., 2004
Rana clamitans	Larvae	96 h			2.2					Roundup Transorb	Howe et al., 2004
Rana clamitans	Embryo	96 h (pH=6)			5.3					Vision (glyphosate-based herbicide containing a 15% (weight:weight) polyethoxylated tallow amine surfactant blend)	Edginton et al., 2004
Rana clamitans	Embryo	96 h (pH=7.5)			4.1					Vision (glyphosate-based herbicide containing a 15% (weight:weight) polyethoxylated tallow amine surfactant blend)	Edginton et al., 2004
Rana clamitans	Larvae	96 h (pH=6)			3.5					Vision (glyphosate-based herbicide containing a 15% (weight:weight) polyethoxylated tallow amine surfactant blend)	Edginton et al., 2004
Rana clamitans	Larvae	96 h (pH=7.5)			1.4					Vision (glyphosate-based herbicide containing a 15% (weight:weight) polyethoxylated tallow amine surfactant blend)	Edginton et al., 2004
Rana clamitans	Larvae	96h			4.22			3.27	3.68	Roundup Original	Fuentes et al., 2011
Rana clamitans	Larvae	24 h			>17.9					Glyphosate Technical	Howe et al., 2004
Rana clamitans	Larvae	96 h			>17.9					Glyphosate Technical	Howe et al., 2004
Rana clamitans	Tadpole	16 d	2.17		1.6275					Roundup®	Relyea, 2005
Rana clamitans	Larvae	96 h			1.4					Roundup Original Max	Relyea and Jones, 2009
Rana clamitans	Larvae	96 h			4.34					Vision (glyphosate, 356 mg acid equivalents (a.e.)/L)	Wojtaszek et al., 2004
Rana clamitans	Larvae	96 h			2.7					Vision (glyphosate, 356 mg acid equivalents (a.e.)/L)	Wojtaszek et al., 2004
Rana clamitans	Larvae	96 h			2.77			1.91	2.37	Roundup WeatherMAX	Fuentes et al., 2011
Rana clamitans	Larvae	96 h			4.22			3.27		Roundup®	Moore et al., 2012
Rana pipiens	Larvae	24 h			3.7					Roundup Original	Howe et al., 2004
Rana pipiens	Larvae	96 h			2.9					Roundup Original	Howe et al., 2004
Rana pipiens	Embryo	24 h			>8					Roundup Original	Howe et al., 2004
Rana pipiens	Embryo	96 h			6.5					Roundup Original	Howe et al., 2004
Rana pipiens	Embryo	96 h (pH=6)			15.1					Vision (glyphosate-based herbicide containing a 15% (weight:weight) polyethoxylated tallow amine surfactant blend)	Edginton et al., 2004
Rana pipiens	Embryo	96 h (pH=7.5)			7.5					Vision (glyphosate-based herbicide containing a 15% (weight:weight) polyethoxylated tallow amine surfactant blend)	Edginton et al., 2004

AMPHIBIANS											
Glyphosate											
Species	Life stage	Test duration	LC50			NOEC			LOEC	Formulation	Reference
			(mg a.i L^{-1})	(mg RU L^{-1})	(mg a.e L^{-1})	(mg a.i L^{-1})	(mg RU L^{-1})	(mg a.e L^{-1})	(mg a.e L^{-1})		
Rana pipiens	Larvae	96 h (pH=6)			1.8					Vision (glyphosate-based herbicide containing a 15% (weight:weight) polyethoxylated tallow amine surfactant blend)	Edginton et al., 2004
Rana pipiens	Larvae	96 h (pH=7.5)			1.1					Vision (glyphosate-based herbicide containing a 15% (weight:weight) polyethoxylated tallow amine surfactant blend)	Edginton et al., 2004
Rana pipiens	Larvae	96 h			11.47					Vision (glyphosate, 356 mg acid equivalents (a.e.)/L)	Wojtaszek et al., 2004
Rana pipiens	Larvae	96 h			4.25					Vision (glyphosate, 356 mg acid equivalents (a.e.)/L)	Wojtaszek et al., 2004
Rana pipiens	Larvae	96 h			1.8			1.29	1.32	Roundup Original	Fuentes et al., 2011
Rana pipiens	Larvae	96 h			1.5					Roundup Original Max	Relyea and Jones, 2009
Rana pipiens	Larvae	96 h			1.8			1.29		Roundup®	Moore et al., 2012
Rana pipiens	Larvae	96 h			2.27			1.65	1.68	Roundup WeatherMAX	Fuentes et al., 2011
Rana sphenocephala	Larvae	96 h			1.33			**0.68**	0.98	Roundup WeatherMAX	Fuentes et al., 2011
Rana sphenocephala	Larvae	96 h			2.05			1.52	1.81	Roundup Original	Fuentes et al., 2011
Rana sylvatica	Larvae	24 h			5.6					Roundup Original	Howe et al., 2004
Rana sylvatica	Larvae	96 h			5.1					Roundup Original	Howe et al., 2004
Rana sylvatica	Embryo	24 h			>8					Roundup Original	Howe et al., 2004
Rana sylvatica	Embryo	96 h			>8					Roundup Original	Howe et al., 2004
Rana sylvatica	Larvae	96 h			1.9					Roundup Original Max	Relyea and Jones, 2009
Rhinella arenarum	Tadpole	6 h			5.62					Roundup Ultra-Max	Lajmanovich et al., 2011
Rhinella arenarum	Tadpole	12 h			3.26					Roundup Ultra-Max	Lajmanovich et al., 2011
Rhinella arenarum	Tadpole	24 h			2.42					Roundup Ultra-Max	Lajmanovich et al., 2011
Rhinella arenarum	Tadpole	48 h			2.42					Roundup Ultra-Max	Lajmanovich et al., 2011
Rhinella arenarum	Tadpole	6 h			49.65					Infosato	Lajmanovich et al., 2011
Rhinella arenarum	Tadpole	12 h			47.25					Infosato	Lajmanovich et al., 2011
Rhinella arenarum	Tadpole	24 h			38.76					Infosato	Lajmanovich et al., 2011
Rhinella arenarum	Tadpole	48 h			38.76					Infosato	Lajmanovich et al., 2011
Rhinella arenarum	Tadpole	6 h			96.87					Glifoglex	Lajmanovich et al., 2011
Rhinella arenarum	Tadpole	12 h			77.52					Glifoglex	Lajmanovich et al., 2011
Rhinella arenarum	Tadpole	24 h			73.77					Glifoglex	Lajmanovich et al., 2011
Rhinella arenarum	Tadpole	48 h			73.77					Glifoglex	Lajmanovich et al., 2011
Rhinella arenarum	Tadpole	6 h			104.33					C-K YUYOS FAV	Lajmanovich et al., 2011
Rhinella arenarum	Tadpole	12 h			84.06					C-K YUYOS FAV	Lajmanovich et al., 2011
Rhinella arenarum	Tadpole	24 h			77.52					C-K YUYOS FAV	Lajmanovich et al., 2011
Rhinella arenarum	Tadpole	48 h			77.52					C-K YUYOS FAV	Lajmanovich et al., 2011
Scinax nasicus	Tadpole	24 h			1.692					GLYFOS (48% glypkosate as isopropylamine salt)	Lajmanovich et al., 2003

AMPHIBIANS											
**Glyphosate**											
**Species**	**Life stage**	**Test duration**	**LC50**			**NOEC**			**LOEC**	**Formulation**	**Reference**
			**(mg a.i L^{-1})**	**(mg RU L^{-1})**	**(mg a.e L^{-1})**	**(mg a.i L^{-1})**	**(mg RU L^{-1})**	**(mg a.e L^{-1})**	**(mg a.e L^{-1})**		
Scinax nasicus	Tadpole	48 h			1.3032					GLYFOS (48% glypkosate as isopropylamine salt)	Lajmanovich et al., 2003
Scinax nasicus	Tadpole	72 h			1.1628					GLYFOS (48% glypkosate as isopropylamine salt)	Lajmanovich et al., 2003
Scinax nasicus	Tadpole	96 h			0.95					GLYFOS (48% glypkosate as isopropylamine salt)	Lajmanovich et al., 2003
Spea bombifrons	Larvae	48 h			2.03					RoundupWeatherMAX	Dinehart et al., 2010
Spea bombifrons	Larvae	216 h			1.99					RoundupWeatherMAX	Dinehart et al., 2010
Spea bombifrons	Larvae	48 h			1.85					RoundupWeatherMAX	Dinehart et al., 2010
Spea bombifrons	Larvae	216 h			1.65					RoundupWeatherMAX	Dinehart et al., 2010
Spea multiplicata	Larvae	48 h			2.3					RoundupWeatherMAX	Dinehart et al., 2010
Spea multiplicata	Larvae	216 h			1.93					RoundupWeatherMAX	Dinehart et al., 2010
Spea multiplicata	Larvae	48 h			2.11					RoundupWeatherMAX	Dinehart et al., 2010
Spea multiplicata	Larvae	216 h			2.11					RoundupWeatherMAX	Dinehart et al., 2010
Xenopus laevis	Embryo	4 d		72	22.3					Roundup®	Perkins, 1997
Xenopus laevis	Embryo	96 h (pH=6)			15.6					Vision (glyphosate-based herbicide containing a 15% (weight:weight) polyethoxylated tallow amine surfactant blend)	Edginton et al., 2004
Xenopus laevis	Embryo	96 h (pH=7.5)			7.9					Vision (glyphosate-based herbicide containing a 15% (weight:weight) polyethoxylated tallow amine surfactant blend)	Edginton et al., 2004
Xenopus laevis	Larvae	96 h (pH=6)			2.1					Vision (glyphosate-based herbicide containing a 15% (weight:weight) polyethoxylated tallow amine surfactant blend)	Edginton et al., 2004
Xenopus laevis	Larvae	96 h (pH=7.5)			0.88					Vision (glyphosate-based herbicide containing a 15% (weight:weight) polyethoxylated tallow amine surfactant blend)	Edginton et al., 2004
**AMPA**											
**No data available**											

IC50: median inhibitory concentration, EC50: median effective concentration, NOEC: no observed effects concentration, LOEC: lowest observed effect concentration, observed effects concentration, RU: Roundup. Bold value indicates lower NOEC for amphibians.

Table S5: Toxicity of Glyphosate and AMPA to aquatic macrophytes.

AQUATIC MACROPHYTES							
**Glyphosate**							
**Species**	**Test Duration**	**IC50 / EC50**		**NOEC**		**Formulation**	**Reference**
		**(mg RU L^{-1})**	**(mg a.e L^{-1})**	**(mg RU L^{-1})**	**(mg a.e L^{-1})**		
Lemna gibba	7 d	15,1	4,7			Roundup®	Perkins, 1997
Lemna gibba	7 d		10			Glyphosate	Perkins, 1997
Lemna gibba	14 d		25,5		16,6	Glyphosate	Malcolm Pirnie, 1987d
Lemna minor	14 d	4,9	1,5			Roundup®	Hartman and Martin, 1984
Lemna minor	14 d			56	17,4	Roundup®	Lockhart et al., 1989
Myriophyllum sibirieum	14 d	3,9	1,2			Roundup®	Perkins, 1997
Myriophyllum sibirieum	14 d		1,6			Glyphosate	Perkins, 1997
Potamogeton pectinatus	14 d			24	**7,4**	Roundup®	Hartman and Martin, 1985
**AMPA**							
**No data available**							

LC50: median lethal concentration, EC50: median effective concentration, NOEC: no observed effects concentration, RU: Roundup. Bold value indicates lower NOEC for aquatic macrophytes.

Table S6: Toxicity of Glyphosate to benthic organisms.

BENTHIC ORGANISMS									
Glyphosate									
**Species**	**Test duration**	**EC50/LC50**			**NOEC**			**Formulation**	**Reference**
		**(mg a.i L^{-1})**	**(mg RU L^{-1})**	**(mg a.e L^{-1})**	**(mg a.i L^{-1})**	**(mg RU L^{-1})**	**(mg a.e L^{-1})**		
Chironomus plumosus	2 d		58,1	18,0				Roundup®	Folmar et al., 1979
Chironomus plumosus	2 d	55		41				Glyphosate (tested as IPA salt)	Folmar et al., 1979
Chironomus riparius	2 d	5600		4200				Glyphosate (tested as IPA salt)	Buhl and Faerber, 1989
Chrionomus tentansd	10 d	>530		398	265		199	Glyphosate (tested as IPA salt)	Beyers, 1993
Tubifex tubifex	28 d					>89	**> 28**	Roundup®	Perkins, 1997

LC50: median lethal concentration, EC50: median effective concentration, NOEC: no observed effects concentration, RU: Roundup. Bold value indicates lower NOEC for aquatic benthic organisms.

References of Supplementary material.

ABC Inc. (1978a) Acute toxicity of technical glyphosate to Daphnia magna. Monsanto unpublished study AB-78-201. Analytical Biochemistry Laboratories, Inc., Columbia, MO. (Cited in Giesy et al. 2000).

ABC Inc. (1978b) Acute toxicity of technical glyphosate to rainbow trout (Salmo gairdnerii). Monsanto unpublished study AB-78-165. Analytical Biochemistry Laboratories, Inc., Columbia, MO. (Cited in Giesy et al. 2000).

ABC Inc. (1978c) Acute toxicity of technical glyphosate to bluegill sunfish (Lepomis macrochirus). Monsanto unpublished study AB-78-123. Analytical Biochemistry Laboratories, Inc., Columbia, MO. (Cited in Giesy et al. 2000).

ABC Inc. (1981a) Acute toxicity of MON 0139 to Daphnia magna. Monsanto unpublished study AB-81-074. Analytical Biochemistry Laboratories, Inc., Columbia, MO. (Cited in Giesy et al. 2000).

ABC Inc. (1981b) Acute toxicity of MON 0139 to bluegill sunfish (Lepomis macrochirus). Monsanto unpublished study AB-81-073. Analytical Biochemistry Laboratories, Inc., Columbia, MO. (Cited in Giesy et al. 2000).

ABC Inc. (1981c) Acute toxicity of MON 0139 to rainbow trout (Salmo gairdneri). Monsanto unpublished study AB-81-072. Analytical Biochemistry Laboratories, Inc., Columbia, MO. (Cited in Giesy et al. 2000).

ABC Inc. (1982a) Dynamic 96-hour acute toxicity of Roundup to bluegill sunfish (Lepomis macrochirus). Monsanto unpublished study AB-82-33. Analytical Biochemistry Laboratories, Inc., Columbia, MO. (Cited in Giesy et al. 2000).

ABC Inc. (1982b) Dynamic 48-hour acute toxicity of Roundup to Gammarus pseudominaeus. Monsanto unpublished study AB-82-035. Analytical Biochemistry Laboratories, Inc., Columbia, MO. (Cited in Giesy et al. 2000).

ABC Inc. (1982c) Dynamic 96-hour acute toxicity of Roundup to rainbow trout (Salmo gairdnerii). Monsanto unpublished study AB-82-034. Analytical Biochemistry Laboratories, Inc., Columbia, MO. (Cited in Giesy et al. 2000).

ABC Inc. (1982d) Chronic toxicity of glyphosate to Daphnia magna under flow-through test conditions. Monsanto unpublished study AB-82-036. Analytical Biochemistry Laboratories, Inc., Columbia, MO. (Cited in Giesy et al. 2000).

ABC Inc. (1989a) Flow-through toxicity of glyphosate to rainbow trout (Salmo gairdnerii) for a 21 day duration period. Monsanto unpublished study AB-89-036. Analytical Biochemistry Laboratories, Inc., Columbia, MO. (Cited in Giesy et al. 2000).

ABC Inc. (1989b) 21 Day prolonged static renewal toxicity of Roundup to Daphnia magna. Monsanto unpublished study AB-89-059. Analytical Biochemistry Laboratories, Inc., Columbia, MO. (Cited in Giesy et al. 2000).

ABC Inc. (1989c) 21 Day prolonged static renewal toxicity of glyphosate technical to Daphnia magna. Monsanto unpublished study AB-89-058. Analytical Biochemistry Laboratories, Inc., Columbia, MO. (Cited in Giesy et al. 2000).

ABC Inc. (1989d) Flow-through toxicity of Roundup to rainbow trout (Salmo gairdnerii) for a 21 day exposure period. Monsanto unpublished study AB-89-037. Analytical Biochemistry Laboratories, Inc., Columbia, MO. (Cited in Giesy et al. 2000).

ABC Inc. (1991a) Acute toxicity of AMPA to Daphnia magna. Monsanto unpublished study AB-90-401. Analytical Biochemistry Laboratories, Inc., Columbia, MO. (Cited in Giesy et al. 2000).

ABC Inc. (1991b) Acute toxicity of AMPA to rainbow trout (Oncorhynchus mykiss). Monsanto unpublished study AB-90-402. Analytical Biochemistry Laboratories, Inc., Columbia, MO. (Cited in Giesy et al. 2000).

Beyers DW (1993) Acute toxicity of Rodea and Valent X-77 to Rio Grande silvery minnow as estimated by surrogate species: Plains minnow, fathead minnow, Hyalella azteca, and Chironomus tentans. US Bureau of Reclamation, Upper Colorado Regional office, Final Report, Feb 12 (Cited in Giesy et al. 2000).

Beyers DW (1995) Acute toxicity of Rodeo® herbicide to Rio Grande silvery minnow as estimated by surrogate species: plains minnow and fathead minnow. Arch Environ Contam Toxicol 29:24-26.

Bidwell JR, Gorrie JR (1995) Acute toxicity of a herbicide to selected frog species. Final report. Prepared for Western Australian Department of Environmental Protection, Westralia Square, 141 St. George's Terrace, Perth, 6000, Australia.

Bionomics (1973) Acute toxicity of Roundup to bluegill (Lepomis macrochirus). Monsanto unpublished study BN-73-078. Bionomics, Wareham, MS. (Cited in Giesy et al. 2000).

Bonnet JL, Bonnemoy F, Dusser M, Bohatier J (2007) Assessment of the potential toxicity of herbicides and their degradation products to nontarget cells using two microorganisms, the bactéria *Vibrio fischeri* and the ciliate *Tetrahymena pyriformis*. Environ. Toxicol. 22(1):78–91.

Bozeman J, Koopman B, Bitton G (1989) Toxicity testing using immobilized algae. Aquat Toxicol 14(4):345-352.

Bringolf RB, Cope WG, Mosher S, Barnhart MC, Shea D (2007) Acute and chronic toxicity of glyphosate compounds to glochidia and juveniles of Lampsilis siliquoidea (Unionidae). Environ. Toxicol. Chem. 26(10):2094–2100.

Buhl KJ, Faerber NL (1989) Acute toxicities of selected herbicides and surfactants to larvae of the midge Chironomus riparius. Arch Environ Contam Toxicol18:530-536.

Christian FA, Jackson RN, Tate TM (1993) Effect of sublethal concentrations of glyphosate and dalapon on protein and aminotransferase activity in Pseudosuccinea columella. Bull Environ Contam Toxicol 51:703-709.

Christy SL, Karlander EP, Parochetti IV (1981) Effects of glyphosate on the growth rate of Chlorella. Weed Sci 29(1):5-7.

Dengler D, Mende P (1994a) Testing of toxic effects of aminomethyl phosphonic acid (AMPA) on the single cell green alga (Scenedesmus subspicatus). Unpublished study XX-93-271. Monsanto Company, St. Louis, MO. (Cited in Giesy et al. 2000).

Dengler D, Mende P (1994b) Testing of toxic effects of the isopropylamine salt of glyphosate acid on the single cell green alga Scenedesmus subspicatus. Unpublished study XX-93-270. Monsanto Company, St. Louis, MO. (Cited in Giesy et al. 2000).

Dinehart SK, Smith LM, McMurry ST, Smith PN, Anderson TA, Haukos DA (2010) Acute and chronic toxicity of Roundup Weathermax (®) and Ignite (®) 280 sl to larval Spea multiplicata and S. bombifrons from the Southern high plains, USA. Environ. Pollut. 158(8):2610–2617.

Edginton AN, Sheridan PM, Stephenson GR, Thompson DG, Boermans HJ (2004) Comparative effects of pH and Vision (®) herbicide on two life stages of four anuran amphibian species. Environ. Toxicol. Chem. 23(4):815–822.

EG & G Bionomics (1975) Chronic toxicity of glyphosate to the fathead minnow (Pimephales promelas Rafinesque). Monsanto unpublished study BN-75-129. EG & G Bionomics, Aquatic Toxicology Laboratory. Wareham, MS. (Cited in Giesy et al. 2000).

EG & G Bionomics (1978) Toxicity of seven test materials to sheepshead minnows, (Cyprinodon variegatus). Monsanto unpublished study BN-78-44B. EG & G Bionomics, Marine Research Laboratory, Pensacola, FL. (Cited in Giesy et al. 2000).

EG & G Bionomics (1980a) Acute toxicity of Roundup to channel catfish (Ictalurus punctatus). Monsanto unpublished study BN-80-076. EG & G Bionomics, Aquatic Toxicology Laboratory, Wareham, MS. (Cited in Giesy et al. 2000).

EG & G Bionomics (1980b) Acute toxicity of Roundup to bluegill (Lepomis macrochirus). Monsanto unpublished study BN-80-075. EG & G Bionomics, Aquatic Toxicology Laboratory, Wareham, MS. (Cited in Giesy et al. 2000).

EG & G Bionomics (1980c) Acute toxicity of Roundup to the rainbow trout (Salmo gairdnerii). Monsanto unpublished study BN-80-074. EG & G Bionomics, Aquatic Toxicology Laboratory, Wareham, MS. (Cited in Giesy et al. 2000).

EG & G Bionomics (1980d) Acute toxicity of Roundup to the fathead minnow (Pimephales promelas). Monsanto unpublished study BN-80-077. EG & G Bionomics, Aquatic Toxicology Laboratory, Wareham, MS. (Cited in Giesy et al. 2000).

EG & G Bionomics (1980e) Acute toxicity of Roundup to the water flea (Daphnia magna). Monsanto unpublished report BN-80-079. EG & G Bionomics, Aquatic Toxicology Laboratory, Wareham, MS. (Cited in Giesy et al. 2000).

EG & G Bionomics (1980f) Acute toxicity of Roundup to the water flea (Daphnia magna) with and without continuous aeration. Monsanto unpublished study BN-80- 181. EG & G Bionomics, Aquatic Toxicology Laboratory, Wareham, MS. (Cited in Giesy et al. 2000).

EG & G Bionomics (1980g) Acute toxicity of Roundup to the rainbow trout (Salrno gairdnerii) with continuous aeration and without aeration. Monsanto unpublished study BN-80-180. EG & G Bionomics, Aquatic Toxicology Laboratory, Wareham, MS. (Cited in Giesy et al. 2000).

Folmar LC, Sanders HO, Julin AM (1979) Toxicity of the herbicide glyphosate and several of its formulations to fish and aquatic invertebrates. Arch. Environ. Contam. Toxicol. 8(3):269–278.

Fuentes L, Moore LJ, Rodgers JH, Bowerman WW, Yarrow GK, Chao WY (2011) Comparative toxicity of two glyphosate formulations (original formulation of Roundup (®) and Roundup Weathermax (®) to six north american larval anurans. Environ. Toxicol. Chem. 30(12):2756–2761.

Gardner SC, Grue CE, Grassley JM, Lenz LA, Lindenauer JM, Seeley ME (1997) Single species algal (Ankistrodesmus) toxicity tests with Rodeo and Garlon 3A. Bull Environ Contam Toxicol 59:492-499.

Giesy JP, Dobson S, Solomon KR (2000) Ecotoxicological risk assessment for Roundup herbicide. Rev Environ Contam Toxicol 167:35–120

Hartman WA, Martin DB (1984) Effect of suspended bentonite clay on the acute toxicity of glyphosate to Daphnia pulex and Lemna minor. Bull Environ Contam Toxicol 33(3):355-361.

Hartman W A, Martin DB (1985) Effects of four agricultural pesticides on Daphnia pulex, Lemna minor, and Potamogeton pectinatus. Bull Environ Contam Toxicol 35(5): 646-651.

Hernando F, Royuela M, Munoz-Rueda A, Gonzalez-Murua C (1989) Effects of glyphosate on the greening process and photosynthetic metabolism in Chlorella pyrenaidosa. J Plant Physiol 134:26-31.

Hess FD (1980) A Chlamydomonas algal bioassay for detecting growth inhibitor herbicides. Weed Sci 28(5):515-520.

Hildebrand LD, Sullivan DS, Sullivan TP (1982) Experimental studies of rainbow trout populations exposed to field applications of Roundup herbicide. Arch Environ Contam Toxicol 11:93-98.

Holck AR, Meek CL (1987) Dose-mortality responses of crawfish and mosquitoes to selected pesticides. J Amer Mosquito Control Assoc 3(3):407-411.

Holdway DA, Dixon DG (1988) Acute toxicity of permethrin or glyphosate pulse exposure to larval white sucker (Catostomus commersoni) and juvenile flagfish (Jordanella floridae) as modified by age and ration level. Environ Toxicol Chern 7:63-68.

Howe CM, Berrill M, Pauli BD, Helbing CC, Werry K, Veldhoen N (2004) Toxicity of glyphosate-based pesticides to four North american frog species. Environ. Toxicol. Chem. 23(8):1928–1938.

HRC (1977) The acute toxicity of glyphosate to harlequin fish (Rasbora heteromorpha). Monsanto unpublished study HU-77-465. Huntingdon Research Centre, Huntingdon, UK. (Cited in Giesy et al. 2000).

Hued AC, Oberhofer S, Bistoni MD (2012) Exposure to a commercial glyphosate formulation (Roundup®) alters normal gill and liver histology and affects male sexual activity of Jenynsia multidentata (Anablepidae, Cyprinodontiformes). Arch. Environ. Contam. Toxicol. 62(1):107–117.

Kreutz LC, Barcellos LJG, Silva TO, Anziliero D, Martins D, Lorenson M, Marteninghe A, Da Silva LB (2008) Acute toxicity test of agricultural pesticides on silver catfish (Rhamdia quelen) fingerlings. Ciencia Rural 38(4):1050–1055.

Lajmanovich RC, Sandoval MT, Peltzer PM (2003) Induction of mortality and malformation in Scinax nasicus tadpoles exposed to glyphosate formulations. Bull. Environ. Contam. Toxicol. 70: 612–618.

Lajmanovich RC, Attademo AM, Peltzer PM, Junges CM, Cabagna MC (2011) Toxicity of four herbicide formulations with glyphosate on Rhinella arenarum (Anura: Bufonidae) tadpoles: B-esterases and glutathione s-transferase inhibitors. Arch. Environ. Contam. Toxicol. 60:681–689.

Langiano VC, Martinez CBR (2008) Toxicity and effects of a glyphosatebased herbicide on the neotropical fish Prochilodus lineatus. Comp. Biochem. Physiol. C: Toxicol. Pharmacol. 147:222–231.

Liong PC, Hamzah WP, Murugan V (1988) Toxicity of some pesticides towards freshwater fishes. Malays Agric J 54(3):147-156.

LISEC (1989) Alga, growth inhibition test. Effect of MON 2139 on the growth of Selenastrum capricomutum. Monsanto unpublished study XX-89-093. LISEC, Study Centre for Ecology and Forestry, Bokrijk, Belgium. (Cited in Giesy et al. 2000).

Lockhart WL, Billeck BN, Baron CL (1989) Bioassays with a floating aquatic plant (Lemna minor) for effects of sprayed and dissolved glyphosate. Hydrobiologia 188/ 189:353-359.

Ma J (2002) Differential sensitivity to 30 herbicides among populations of two green algae Scenedesmus obliquus and Chlorella pyrenoidosa. Bull. Environ. Contam. Toxicol. 68:275–281.

Malcolm Pirnie (1987a) The toxicity of glyphosate technical to Anabaena flos-aquae. Monsanto unpublished study XX-88-415. Malcolm Pimie Inc., White Plains, NY. (Cited in Giesy et al. 2000).

Malcolm Pirnie (1987b) The toxicity of glyphosate technical to Skeletonema costatum. Monsanto unpublished study XX-88-414. Malcolm Pimie Inc., White Plains, NY. (Cited in Giesy et al. 2000).

Malcolm Pirnie (1987c) The toxicity of glyphosate technical to Navicula pelliculosa. Monsanto unpublished study XX-88-413. Malcolm Pimie Inc., White Plains, NY. (Cited in Giesy et al. 2000).

Malcolm Pirnie (1987d) The toxicity of glyphosate technical to Lemna gibba. Monsanto unpublished study XX-88-416. Malcolm Pimie Inc., White Plains, NY. (Cited in Giesy et al. 2000).

Mann RM, Bidwell JR (1999) The toxicity of glyphosate and several glyphosate formulations to four species of southwestern Australian frogs. Arch Environ Con tam Toxicol 36:193-199.

Maule A, Wright SJL (1984) Herbicide effects on the populations growth of some green algae and cyanobacteria. J Appl Bacteriol 57:369-379.

Mayer FL, Ellersieck MR (1986) Manual of acute toxicity: interpretation and data base for 410 chemicals and 66 species of freshwater animals. United States Department of the Interior, Fish and Wildlife Service Resource Publication 160. Washington, DC.

Mitchell DG, Chapman PM, Long TJ (1987) Acute toxicity of Roundup and Rodeo herbicides to rainbow trout, chinook, and coho salmon. Bull Environ Con tam Toxicol 39(6): 1028-1035.

Moore LJ, Fuentes L, Rodgers JH Jr, Bowerman WW, Yarrow GK, Chao WY, Bridges WC, Jr (2012) Relative toxicity of the components of the original formulation of Roundup® to five north american anurans. Ecotoxicol. Environ. Saf. 78:128–133.

Morgan JD, Vigers GA, Farrell AP, Janz DM, Manville JF (1991) Acute avoidance reactions and behavioral responses of juvenile rainbow trout (Oncorhynchus mykiss) to Garlon 4, Garlon 3ª and Vision herbicides. Environ Toxicol Chern 10:73-79.

Morgan MJ, Kiceniuk JW (1992) Response of rainbow trout to a two month exposure to Vision, a glyphosate herbicide. Bull Environ Contam Toxicol 48:772-780.

NATEC (1990) Glyphosate: growth inhibition test with algae according to OECD-guideline 201. Monsanto unpublished study XX-90-523. Study NA89 9654. NATEC Institut fur Naturwissenschaftlich Technische Dienste GmbH, Hamburg, Germany. (Cited in Giesy et al. 2000).

Perkins MJ (1997) Effects of two formulations of glyphosate and triclopyr on four nontarget aquatic species: Xenopus laevis, Myriophyllum sibiricum, Lemna gibba, and Tubifex tUbifex. M.Sc. thesis. University of Guelph, Guelph, Ontario, Canada.

Relyea RA (2005) The lethal impacts of Roundup and predatory stress on six species of North american tadpoles. Arch. Environ. Contam. Toxicol. 48(3):351–357.

Relyea RA, Jones DK (2009) The toxicity of Roundup OriginalMax® to 13 species of larval amphibians. Environ. Toxicol. Chem. 28(9):2004–2008.

Saenz ME, Di Marzio WD, Alberdi JL, del Carmen Tortorelli M (1997) Effects of technical grade and a commercial formulation of glyphosate on algal population growth. Bull Environ Contam Toxicol 59(4):638-644.

Servizi JA, Gordon RW, Marten DW (1987) Acute toxicity of Garlon 4 and Roundup herbicides to salmon, Daphnia, and trout. Bull Environ Contam Toxicol 39(1):15-22.

Sun F (1987) Evaluating acute toxicity of pesticides to aquatic organisms carp, mosquitofish and daphnids. Plant Prot Bull 29(4):385-396.

Thomas MW, Judy BM, Lower WR, Krause GF, Sutton WW (1990) Time-dependent toxicity assessment of herbicide contaminated soil using the green alga Selenastrum capricornutum. In: Wand W, Gorusch JW, Lower WR (eds) Plants for Toxicity Assessment. ASTM STP 1091. American Society for Testing and Materials, Philadelphia, p 235.

Thompson J (1989) Chronic effects of sub-lethal levels of dalapon, glyphosate, and 2,4- D amine on the lymnaeid snails Pseudosuccinea columella and Fossaria cubensis. M.S. thesis, Southern University Library, Baton Rouge, LA. (Cited in Christian et al. 1993).

Tooby TE, Lucey J, Stott B (1980) The tolerance of grass carp, Ctenopharyngodon idella Val., to aquatic herbicides. J Fish BioI 16:591-597.

Tsui MTK, Chu LM (2003) Aquatic toxicity of glyphosate-based formulations: Comparison between different organisms and the effects of environmental factors. Chemosphere 52(7):1189–1197.

Vendrell E, de Barreda Ferraz DG, Sabater C, Carrasco JM (2009) Effect of glyphosate on growth of four freshwater species of phytoplankton: A microplate bioassay. Bull. Environ. Contam. Toxicol. 82(5):538–542.

Wan MT, Watts RG, Moul OJ (1989) Effects of different dilution water types on the acute toxicity to juvenile Pacific salmonids and rainbow trout of glyphosate and its formulated products. Bull Environ Contam Toxicol 43(3):378-385.

WHO (World Health Organization) (1994) Glyphosate: environmental health criteria 159. World Health Organization, Geneva, Switzerland.

Wojtaszek BF, Staznik B, Chartrand DT, Stephenson GR, Thompson DG (2004) Effects of Vision® herbicide on mortality, avoidance response, and growth of amphibian larvae in two forest wetlands. Environ. Toxicol. Chem. 23(4):832–842.
